# Transformer-based human-motion forecasting coupled with safe reinforcement learning for telepresence robot co-navigation

**DOI:** 10.3389/fnbot.2025.1697518

**Published:** 2026-02-02

**Authors:** Heba G. Mohamed, Muhammad Nasir Khan, Fawad Naseer, Muhammad Tahir, Mohsin Jamil

**Affiliations:** 1 Department of Electrical Engineering, College of Engineering, Princess Nourah Bint Abdulrahman University, Riyadh, Saudi Arabia; 2 Department of Electrical Engineering, Government College University Lahore, Lahore, Pakistan; 3 Department of Computer Science and Software Engineering, Beaconhouse International College, Faisalabad, Pakistan; 4 School of Computer Science and Mathematics, Faculty of Engineering, Liverpool John Moores University, Liverpool, United Kingdom; 5 Department of Computer Software Engineering, Sir Syed University of Engineering and Technology, Karachi, Pakistan; 6 Department of Engineering, Brock University, St. Catharines, ON, Canada

**Keywords:** anticipatory perception, control barrier functions (CBF), crowd-aware navigation, healthcare environments, human–robot co-navigation, safe reinforcement learning, telepresence robots (TPRs), transformer-based motion forecasting

## Abstract

**Introduction:**

Telepresence robots (TPRs) must co-navigate with humans in constrained hospital environments, where safety depends on anticipating rather than merely reacting to human motion. Existing approaches rarely integrate short-horizon human-motion forecasting with safety-constrained control, which reduces robustness in dense corridors and ward bays. This study addresses this gap by evaluating an anticipatory, safety-aware co-navigation framework for TPRs.

**Methods:**

We developed a modular framework that couples a lightweight transformer-based forecaster that predicts multi-agent trajectories under occlusion with a safe reinforcement learning (RL) controller. The forecaster produces short-term distributions over pedestrian states that are embedded into the RL policy state and cost as risk-aware occupancy features. Safety is enforced via constrained policy optimization augmented by a run-time control barrier function (CBF) shield that filters unsafe actions. We benchmarked the approach against a social-force or dynamic window approach (DWA), an attention-based crowd-RL policy, and model predictive control (MPC) with CBF. Experiments were conducted across two hospital-like benchmarks (a crowded corridor and a four-bed ward), totaling 2,400 episodes. Outcomes included task success, collision count, minimum human–robot clearance, near-miss events ( ≤ 0.3 m), time-to-goal, CBF violations, and ablations removing forecasting and the CBF shield.

**Results:**

Relative to the best-performing baseline, the proposed method improved task success by 21.6% and reduced collisions by 47.3%. Median minimum human–robot clearance increased by 0.19 m, and near-miss events decreased by 38.5%. Time-to-goal was maintained within +2.7% of MPC+CBF while incurring zero CBF violations under the shield. Ablation studies showed that removing forecasting degraded success by 14.2%, whereas removing the CBF shield increased constraint breaches from 0% to 6.1% of steps.

**Discussion:**

Anticipatory perception combined with Safe-RL yields substantially safer and more reliable telepresence co-navigation in human-dense clinical layouts without sacrificing efficiency. The framework is modular, enabling alternative forecasters and safety shields. Limitations include sensitivity to forecast drift during abrupt changes in crowd flow. Future work will explore on-device adaptation, shared-autonomy overlays to incorporate operator intent, and prospective evaluations in live hospital workflows.

## 1 Introduction

Telepresence robots (TPRs) are increasingly deployed to extend clinical reach and sustain social connections in hospitals and long-term care (LTC) settings. However, navigation in people-dense wards and narrow corridors remains a critical barrier to reliable uptake. Recent clinical and translational studies show that TPRs can reduce caregiver burden and resident loneliness and help maintain care continuity, underscoring their potential value in real services (e.g., mixed-methods and pre–post trials in LTC) (Hung et al., 2025; Hu et al., 2025). At the same time, operational trials in healthcare highlight the practical challenges of moving a TPR safely through dynamic, cluttered clinical spaces, where visibility is partial and human motion is heterogeneous (Leoste et al., 2024). These reports converge on a need for navigation that is not merely reactive but anticipatory and safety-constrained in real time.

A large body of work in socially aware robot navigation formalizes human–robot co-navigation norms, maintaining comfortable distances, respecting implicit right-of-way, and negotiating bottlenecks. Most algorithms still plan with short-horizon, history-based predictors or handcrafted interaction models. Recent surveys in leading robotics venues document the field's progress and open challenges, including reliable forecasting under occlusion and principled safety handling in crowds (Mavrogiannis et al., 2023; Singamaneni et al., 2024). These reviews specifically call for tighter coupling between the perception of future human motion and the decision layers that guarantee safety, especially in constrained indoor spaces such as hospital wards.

Concurrently, human-motion forecasting has advanced with transformer architectures that model long-range temporal dependencies and multi-joint correlations. Journal reports demonstrate that attention-based predictors can deliver accurate, real-time short- and mid-horizon motion predictions suitable for on-robot deployment (Yunus et al., 2023) and for collaborative tasks that benefit from anticipating human intent (Laplaza et al., 2024). However, these perception modules are rarely integrated into closed-loop navigation with formal safety guarantees; in healthcare corridors, even small forecast errors or sudden flow changes can precipitate unsafe proximity, unless the control layer is explicitly safety-aware. These properties align well with hospital and LTC ward navigation, where flows of staff and residents follow relatively structured routines (e.g., rounds, mealtimes, therapy sessions), motion is often slower and assisted (walkers, wheelchairs), and occlusions from curtains, furniture, and equipment are frequent. In such settings, an attention-based forecaster that jointly reasons over multiple agents and local geometry can better anticipate near-future occupancy around a telepresence robot, enabling it to preserve comfortable clearances for vulnerable residents and staff while respecting narrow corridors and multi-bed bays. Empirical studies already demonstrate that mobile telepresence robots are increasingly used in LTC and healthcare facilities. Still, navigation and proxemics remain practical barriers to routine deployment, which further motivates an anticipatory, transformer-based forecaster in this context.

Safety-critical control for robots increasingly leverages safety filters, methods that project candidate actions onto sets known to satisfy constraints at run time. Control barrier functions (CBFs) provide a principled framework to maintain forward invariance of a “safe set,” and recent journal-level tutorials and surveys consolidate their theory and practice for autonomous systems (Hsu et al., 2024; Li et al., 2023). These works emphasize that safety filtering can complement high-performance planners or learned policies, but also warn about conservatism and feasibility under sensing uncertainty—precisely the regime faced by a TPR moving among people with intermittent occlusions.

In parallel, safe reinforcement learning (Safe-RL) refers to reinforcement-learning methods that explicitly encode safety through constraints or risk measures in the objective; this area has matured from conceptual proposals to consolidated frameworks with policy-level constraints and risk-aware objectives. A review synthesizes methods and theory (e.g., constrained Markov decision processes (CMDPs), Lagrangian approaches, safety layers) and catalogs open issues in deploying Safe-RL in real-world robotics (Gu et al., 2024). Complementary work surveys verification and assurance for deep RL policies, offering analysis tools that can be combined with online safety filters (Landers and Doryab, 2023). Despite this progress, the literature still lacks demonstrations that explicitly fuse learned human-motion forecasts with Safe-RL policies and run-time CBF safety shields for TPR co-navigation in healthcare.

**Figure 1:**
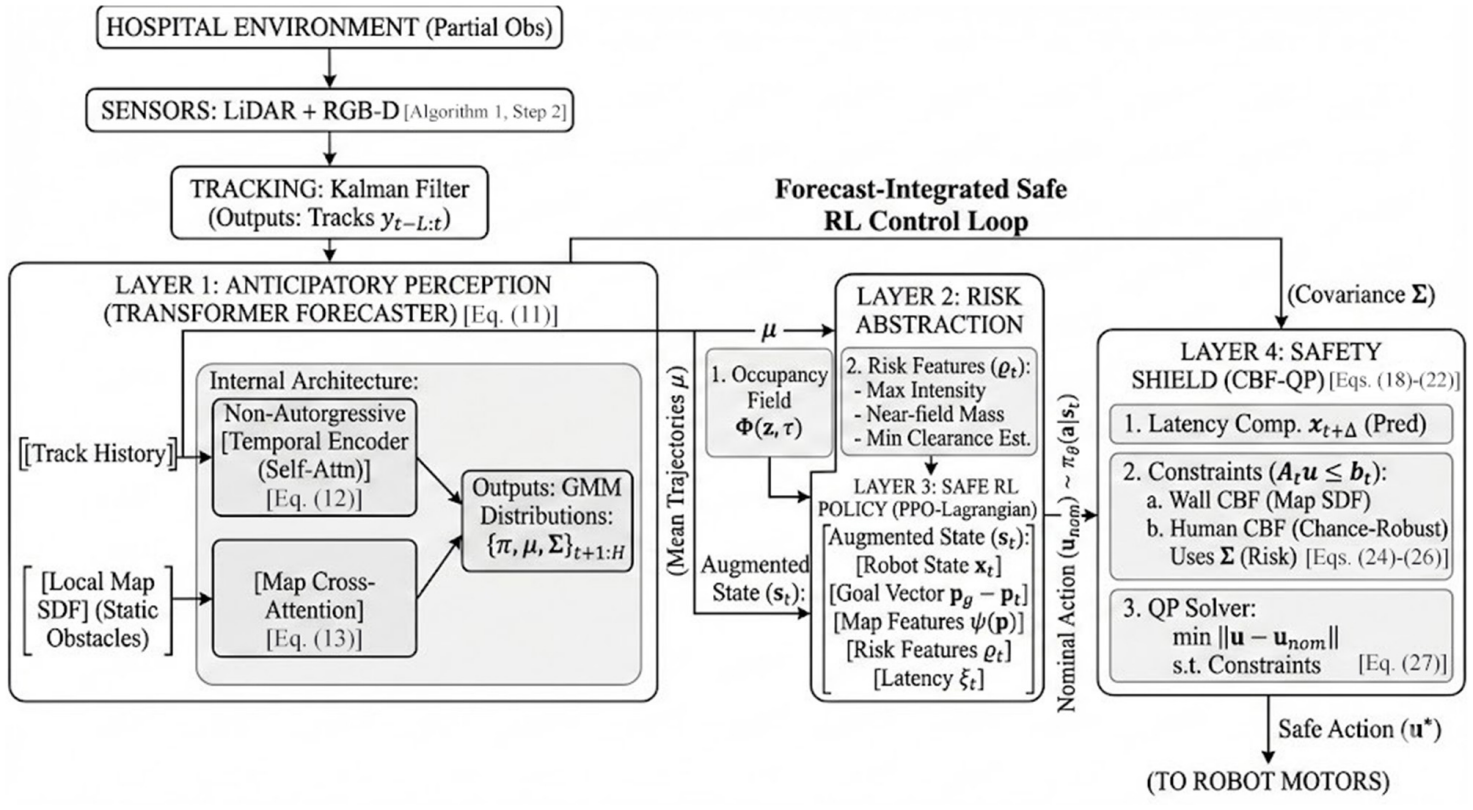
System architecture of the proposed telepresence co-navigation stack. Sensor and map inputs (RGB-D/LiDAR, odometry, signed-distance field) feed a transformer-based human-motion forecaster; its short-horizon multi-agent distributions are converted into occupancy and risk features and combined with robot and latency states to form the Safe-RL policy input; a CBF-based QP shield then minimally adjusts the policy action before low-level control of the telepresence robot.

This study proposes an integrated framework (Figure 1) that (i) learns anticipatory, transformer-based pedestrian-trajectory distributions conditioned on local map structure and occlusion, (ii) injects these distributions into a Safe-RL policy via risk-aware occupancy features, and (iii) enforces hard safety at execution through a CBF-based quadratic-program (QP) “shield” that minimally modifies the policy's action only when necessary. The framework is evaluated in two hospital-like benchmarks—a crowded corridor and a four-bed ward—and is compared against strong baselines spanning social-force/DWA planning, attention-based crowd-RL, and MPC+CBF. The study's contributions are threefold:

1. A problem formulation and modular forecast → policy → safety-filter architecture for telepresence robot co-navigation in hospital- and LTC-style wards, which couples transformer-based human-motion forecasting, Safe-RL, and a discrete-time CBF safety shield under occlusion and latency.
2. A forecast-aware Safe-RL and CBF design that converts short-horizon multi-agent distributions into risk features and chance-robust clearance constraints, using CMDP with Conditional Value-at-Risk (CVaR)-style costs and dual-updated safety budgets.
3. A simulation-based evaluation protocol and dataset for telepresence co-navigation in two hospital-like benchmarks (corridor and four-bed ward), including comparisons to strong social-navigation baselines (ORCA, DWA, PPO, MPC+CBF), ablations, and latency/crowding-sensitivity analyses.

Telepresence robots in hospitals and LTC wards require anticipation under partial observability while operating under tight real-time constraints on embedded compute. We therefore adopt a compact transformer forecaster that can model multi-agent interactions and map-conditioned motion (e.g., doors, curtains, bottlenecks) while maintaining low inference latency at the control frequency. This design targets common LTC traffic patterns—slow, assisted motion and frequent occlusions—so that predictive uncertainty can be used downstream for safety-constrained decision-making.

We do not claim that this architecture addresses all sub-domains of human–robot interaction; our scope is explicitly mobile telepresence navigation in hospital and LTC wards, and other HRI settings (e.g., manipulation-centric or non-navigational interactions) lie outside the remit of this study. By situating the study within documented healthcare use-cases and constraints and by grounding the proposed methods in recent advances in social navigation, motion forecasting, safety filtering, and Safe-RL, the study aims to provide both a practically relevant and methodologically rigorous step toward safer, more reliable telepresence co-navigation in clinical environments.

The remainder of this manuscript is organized as follows. Section 2 reviews related work on human-motion forecasting, shared autonomy, Safe-RL, and control barrier functions; Section 3 formalizes the co-navigation problem, dynamics, and safety constraints; Section 4 details the proposed method—transformer-based forecasting, risk-aware RL, the CBF safety shield, and the end-to-end control cycle. Section 5 describes the experimental setup (ward layout, datasets/simulation, baselines, metrics, and protocols); Section 6 reports results with ablations and sensitivity analyses; Section 7 discusses implications, limitations, and generalizability; Section 8 concludes and outlines future work.

## 2 Literature review

Evidence from LTC, hospital, and home-care contexts indicates that mobile telepresence can improve communication, accelerate specialist access, and support person-centered care while raising persistent concerns about workflow integration, privacy, and acceptance by staff and families (Ren et al., 2024; Wang et al., 2022; Teng et al., 2022). Reviews and qualitative studies highlight gaps in robust navigation and autonomy in busy wards and corridors, limited support for co-navigation with staff and visitors, and the need for safety-assured autonomy under uncertain human motion. These limitations motivate technical advances in human-aware forecasting and safety-critical shared autonomy tailored to clinical layouts.

Over the past five years, surveys have consolidated requirements for social navigation—physical and perceived safety, legibility, naturalness, and compliance with social norms—while documenting the lack of standardized evaluation and hospital-specific benchmarks (Gao and Huang, 2022; Karwowski et al., 2024). At the planner level, widely used baselines include the dynamic window approach (DWA) for reactive local collision avoidance (Fox et al., 1997) and Optimal Reciprocal Collision Avoidance (ORCA) for multi-agent crowd navigation (van den Berg et al., 2011), often combined with social-force or potential-field heuristics in hospital-like simulations (Berg et al., 2010). Empirical proxemics studies quantify comfortable passing distances and personal-space envelopes, which expand with robot speed and scenario (Neggers et al., 2021, 2022). Algorithmic works increasingly embed proxemics into planners, but generalization across densities and group structures remains fragile (Lu et al., 2022; Zhang and Feng, 2023). Collectively, these findings argue for navigation stacks that explicitly model human motion and uncertainty and expose safety-aware blending with human operators.

Transformer variants now dominate pedestrian and agent prediction in traffic and crowd settings, offering non-autoregressive decoding, social-graph attention, and multi-modal prediction (Chen et al., 2023; Shoman et al., 2024; He et al., 2023; Yao et al., 2022; Sun et al., 2023; Liu et al., 2023). Nonetheless, surveys and benchmarks highlight sensitivity to domain shift (site-specific behaviors), long-horizon degradation, and uncertainty calibration—issues that are acute in hospitals where flows are episodic (e.g., shift changes) and space is constrained (Rudenko et al., 2020). This motivates coupling learned forecasting with an online safety layer that can accommodate distributional shifts while preserving social comfort.

Recent tutorials and surveys codify CBFs as forward-invariance constraints that render safety sets robustly invariant when combined with control Lyapunov terms or model predictive control (MPC) (Garg et al., 2024). Emerging variants integrate CBFs with MPC for dynamic obstacle avoidance and feasibility recovery, and begin to address real-time operation on mobile robots (Desai and Ghaffari, 2022; Li et al., 2022; Shayan et al., 2025). These methods offer crisp safety certificates but require reliable state and obstacle estimates and can be conservative without learned intent or forecasting.

Closest to our setting, Samavi et al. introduce SICNav-Diffusion, which combines diffusion-based joint human-trajectory prediction with a bilevel MPC formulation that refines both robot plans and human predictions for safe crowd navigation. In parallel, Mohamed, Ali, and Liu propose a chance-constrained sampling-based MPC (C^2^U-MPPI) that leverages unscented sampling and probabilistic chance constraints to achieve robust collision avoidance in uncertain dynamic environments (Mohamed et al., 2025; Samavi et al., 2025). These works tightly couple probabilistic prediction or uncertainty-aware constraints with MPC-style controllers, but they do not employ Safe-RL or CBF shields, nor do they target telepresence co-navigation in hospital wards; in contrast, our framework uses transformer-based forecasts as belief features within a CMDP-based Safe-RL policy, wrapped by a modular CBF safety filter and evaluated in clinically motivated layouts.

Surveys across robotics and autonomy trace a trend toward constrained MDPs, shielded and predictive safety filters, and risk-sensitive objectives to maintain constraints during learning and deployment (Brunke et al., 2022; Karwowski et al., 2024; Brescia et al., 2023). Despite progress, reviews identify gaps in on-policy safety during exploration, tight guarantees under perception and model uncertainty, and sample efficiency in the presence of rare but critical events—conditions common in hospital corridors. Combining Safe-RL with CBF- or MPC-based certificates is repeatedly recommended to achieve both adaptability and formal safety.

Journal studies in teleoperation and assistive settings converge on adaptive authority allocation via intent prediction (gaze, EMG, motion cues), with user-study evidence that transparency and assistance timing affect agreement and satisfaction (Fuchs and Belardinelli, 2021; Gottardi et al., 2022; Backman et al., 2023; Bowman et al., 2024; Yousefi et al., 2025). However, most systems assume low-dynamic scenes and do not fuse crowd motion forecasts with certified safety layers when blending user and autonomy inputs—a key limitation for telepresence co-navigation in wards and corridors.

The literature offers (i) clinically motivated requirements and adoption barriers, (ii) strong but environment-sensitive transformer forecasters, (iii) formal safety layers via CBF/MPC, (iv) Safe-RL for adaptive policies, and (v) shared-autonomy mechanisms for authority blending. However, a unified pipeline that couples transformer-based human-motion forecasting with Safe-RL policies under an online CBF/MPC safety filter for telepresence co-navigation in hospitals—and evaluates performance using social-comfort and safety metrics from social-navigation standards—has received limited attention. The proposed study targets this integration and clinical evaluation gap.

## 3 Problem formulation

The study formalized the telepresence co-navigation in clinical layouts as a constrained, risk-sensitive decision process with stochastic human dynamics, partial observability, and a run-time safety filter.

### 3.1 Notation and symbols

This section proceeds from a formal problem definition (POMDP and CMDP) to the concrete quantities used by the learning and control modules. Each subsection introduces symbols locally and connects them explicitly to the algorithmic components described later in Section 4. Table 1 summarizes all symbols used throughout Sections 3 and 4. To avoid ambiguity, each symbol is used with a single, consistent meaning across the study. For clarity, we use *x*_*t*_ exclusively for the robot state and *Y*_*t*_ for the joint human state throughout Sections 3 and 4.

**Table 1:** Notation and symbols.

**Study**	**Meaning**
*t*	Discrete-time index
Δ*t*	Control sampling interval
*x*_*t*_	Robot state at time *t*
*u*_*t*_	Control action applied at time *t*
$x_{t}^{H}$	Joint human state
*b*_*t*_	Belief over joint human states
*s*_*t*_	Augmented MDP state observed by the policy
π	Stochastic policy with parameters θ
γ_*disc*_	RL discount factor
γ_*cbf*_	CBF decay parameter
*h*(·)	Control barrier function
λ_*k*_	Dual variable for constraint *k*
λ_max_	Dual clipping threshold
ρ_α_(·)	CVaR risk functional
Δ_*sens*_	Sensing-to-actuation latency
Δ_*net*_	Network latency
Φ(·)	Feature-embedding map
δ*a*_*t*_	Shield intervention vector

### 3.2 Environment, agents, and map geometry

Let the hospital layout be a compact set $M\subset {\mathbb{R}}^{2}$ with free space F=M\O, where O is the union of static obstacles (walls, beds, trolleys). The signed-distance field (SDF) $d_{O}:F\to {\mathbb{R}}_{>0}$ gives the Euclidean distance to ∂O

The TPR is a differential-drive platform with unicycle dynamics. Pedestrians are modeled as disk agents.

- Robot state $x_{t}={\left[p_{t}^{⊤},\theta_{t},\;v_{t}\right]}^{⊤}\in {\mathbb{R}}^{4}$with position${\;p}_{t}={\left[x_{t},\;Y_{t}\right]}^{⊤}$, yaw θ_*t*_ and linear speed *v*_*t*_.
- Control *u*_*t*_ = [*a*_*t*_, ω_*t*_], longitudinal acceleration *a*_*t*_ and yaw rate ω_*t*_.
- Continuous-time dynamics:

$$\dot{x}=f(x,u)=[\begin{array}{c} v\cos\theta \\ v\sin\theta \\ \omega \\ a \end{array}],\;x(0)=x_{0}$$ (1)

- Discrete-time dynamics under zero-order hold (ZOH) with sampling step Δ*t*:

$$\begin{array}{l} x_{t+1}=x_{t}+\Delta t\;f\left(x_{t},u_{t}\right)+w_{t}W\sim \end{array}$$ (2)

ZOH assumes the control input remains constant over each sampling interval Δ*t*, matching the low-level telepresence controller that updates commands at 30–50 Hz.

There are *N*_*t*_ pedestrians at time *t*, indexed by *i*∈ {1, …, *N*_*t*_} with positions $Y_{t}^{i}F\in$. The goal region is = {*p*−||*p*−*p*_*goal*_|| ≤ *r*_*goal*_ }.

**Goal region (functional form):** The goal set is a closed Euclidean ball centered at *p*_*goal*_,


$$\begin{array}{l} G≜\;\left\{pF\in :∥p-p_{goal}∥\leq r_{goal}\right\}. \end{array}$$

**Properties:** G is compact, convex, and closed; its indicator I{p∈G} is discontinuous at the boundary, but the distance-to-goal term used in the stage cost is smooth everywhere except at the goal center.

We define the static safety set as follows:


$$\begin{array}{l} S_{\text{stat}}=\left\{x\in {\mathbb{R}}^{4}:d_{O}\left(p\right)\geq R_{\text{wall}}\right\} \end{array}$$ (3)

which consists of all robot states whose position *p* maintains at least a radius *R*_wall_ from static obstacles encoded by the SDF $d_{O}\left(\cdot \right)$, with comfort radius *R*_wall_> 0.

### 3.3 Observation, partial observability, and belief

Sensors (LiDAR/camera) provide detections *z*_*t*_ within a visibility region $V_{t}F\subset$ (accounting for occlusions by walls and equipment). The co-navigation problem is modeled as a partially observable Markov decision process (POMDP) (Kaelbling et al., 1998), with belief *b*_*t*_ over the joint human state $Y_{t}≜{\left\{Y_{t}^{i}\right\}}_{i=1}^{N_{t}}$, where $Y_{t}^{i}$ denotes the state (e.g., 2D position, optionally velocity) of pedestrian *i* at time *t*.

Let *z*_*t*_ denote the set of sensor detections at time *t* (e.g., tracked 2D positions from LiDAR/RGB-D) observed within the visibility region $V_{t}F\subset$. The human-motion model is τ(*Y*_*t*+1_∣*Y*_*t*_), i.e., the transition density of the joint human state. The observation likelihood is $O\left(z_{t}|Y_{t},V_{t}\right)$, which accounts for occlusions and partial observability through $V_{t}$. We use *b*_*t*_(*Y*_*t*_) to denote the belief distribution over joint human states conditioned on the observation history.

- Observation model: $z_{t}O\sim \left(\cdot |Y_{t},V_{t}\right)$, where $V_{t}$ encodes occlusion-aware visibility in the ward layout.

**Observation likelihood (functional form):** We assume a conditionally independent detection model over visible pedestrians. Let $z_{t}={\left\{z_{t}^{i}\right\}}_{i\in I_{t}^{vis}}$ denote the set of detected 2D pedestrian positions at time *t*, where $I_{t}^{vis}\;$indexes agents currently visible inside $V_{t}$. For each visible agent *i*, we assume


$$\begin{array}{l} z_{t}^{i}=y_{t}^{i}+\nu_{t}^{i},\nu_{t}^{i}N\sim \left(0,\Sigma_{z}\right), \end{array}$$

and missing detections for occluded agents $i\notin I_{t}^{vis}$ are handled via a binary visibility mask induced by $V_{t}$. The resulting likelihood is


$$\begin{array}{l} O\left(z_{t}|Y_{t},V_{t}\right)=\underset{i\in I_{t}^{vis}}{\prod }N\left(z_{t}^{i};y_{t}^{i},\Sigma_{z}\right). \end{array}$$

**Properties:** For visible agents, O is smooth (infinitely differentiable) in $y_{t}^{i}$ and bounded for any fixed Σ_*z*_≻0; occlusions enter only through the mask $I_{t}^{vis}$, yielding a piecewise-smooth likelihood as the visibility set changes.

- Belief evolution over joint human states follows the belief update (Bayes filter) recursion:

$$\begin{array}{l} b_{t+1}\left(Y_{t+1}\right)\propto \int \underset{\text{predict}}{\underset{︸}{\tau \left(Y_{t+1}|Y_{t}\right)b_{t}\left(Y_{t}\right)dY_{t}}}\cdot \underset{\text{correct}}{\underset{︸}{O\left(z_{t+1}|Y_{t+1},V_{t+1}\right)}} \end{array}$$ (4)

Here, the prediction term propagates the prior belief through the motion model τ, and the correction term incorporates the latest observation *z*_*t*+1_ under the visibility constraints $V_{t+\;1}$.

In practice, we do not maintain an explicit grid-based belief over joint human states; instead, the transformer forecaster (Section 4.1) implements the predictive step of this Bayes filter by mapping tracked detections and occlusion-aware map context into short-horizon distributions over pedestrian motion. These distributions serve as a compact belief summary fed to the Safe-RL controller and CBF shield.

To enable tractable control, the proposed formulation embeds a forecast-based summary of *b*_*t*_ into an augmented MDP state *s*_*t*_.

### 3.4 Objective, constraints, and task success

Define a finite horizon *T*. Let *c*(*x*_*t*_, *u*_*t*_; *Y*_*t*_) be an instantaneous task cost and *g*_*j*_(*x*_*t*_, *u*_*t*_; *Y*_*t*_) ≤ 0 constraint functions (safety/comfort), *j* = 1, …, *J*.

- Progress: *c*_*prog*_(*x*_*t*_) = ||*p*_*t*_−*p*_*goal*_ ||_2_.
- Smoothness: $c_{sm}\left(u_{t}\right)=\lambda_{v}a_{t}^{2}+\;\lambda_{\omega }\omega_{t}^{2}$.
- To model social comfort, we define the cost:

$$\begin{array}{l} c_{soc}\left(x_{t};Y_{t}\right)=\overset{N_{t}}{\underset{i=1}{\sum }}\phi \left(||p_{t}-Y_{t}^{i}|{|}_{2}\right) \end{array}$$ (5)

which penalizes proximity between the robot position *p*_*t*_ and nearby humans $Y_{t}^{i}$, thereby encouraging socially compliant navigation behavior. We use $\phi \left(r\right)=\max{\left(0,R_{\text{comfort}}-r\;\right)}^{2}.$

**Social-comfort shaping (functional form and properties):** The hinge-quadratic form $\phi \left(r\right)=\max{\left(0,R_{\text{comfort}}-r\right)}^{2}$ penalizes only when the robot enters a comfort radius *R*_comfort_ while assigning zero cost outside this zone. This ϕ(·) is non-negative, continuous, and piecewise-smooth (differentiable everywhere except at *r* = *R*_comfort_); it is convex for *r* ≤ *R*_comfort_ and has a bounded gradient for any bounded workspace and bounded robot–human distances.

The total per-step cost is defined as


$$\begin{array}{l} c\left(x_{t},u_{t};Y_{t}\right)={\alpha_{cost}c}_{\text{prog}}+{\beta_{cost}c}_{\text{sm}}+{\delta_{cost}c}_{\text{soc}}. \end{array}$$ (6)

This combines progress toward the goal, motion smoothness, and social comfort through weighted terms α_*cost*_, β_*cost*_, and δ_*cost*_, where α_*cost*_, β_*cost*_, δ_*cost*_≥0 weight progress-to-goal, smoothness, and social-comfort costs, respectively (distinct from the CVaR confidence parameter α in Equation 9).

**Dynamic human–robot safety constraint**. We model each pedestrian *i* as a disk of radius *r*_hum_ and the robot footprint as a disk of radius *r*_rob_. Let


$$\begin{array}{l} r_{\text{safe}}≜R_{\text{safe}}+r_{\text{rob}}+r_{\text{hum}}+r_{\text{buf}}, \end{array}$$

where *R*_safe_ is the nominal interpersonal comfort margin and *r*_buf_ is an additional robustness buffer. The hard safety constraint with respect to pedestrian *i* is then


$$\begin{array}{l} g_{i}\left(x_{t};Y_{t}\right)≜r_{safe}^{2}-{∥p_{t}-Y_{t}^{i}∥}^{2}\leq 0,i=1,\ldots ,N_{t} \end{array}$$ (7)

This definition makes *g*_*i*_(·) a signed safety margin: it is non-positive when the robot remains outside the safe disk around each pedestrian.

Static wall safety is enforced through the constraint:


$$\begin{array}{l} g_{\text{wall}}\left(x_{t}\right):=R_{\text{wall}}-d_{O}\left(p_{t}\right)\leq 0. \end{array}$$ (8)

This ensures that the robot remains outside a forbidden margin around walls and other static obstacles at all times.

Goal: success if $p_{T}\in \;G;$ failure on any *g*_*j*_>0 or timeout.

Let the cumulative task cost be $Z=\overset{T-1}{\underset{t=0}{\sum }}c\left(x_{t},u_{t};Y_{t}\right).$ For α∈(0, 1), the CVaR objective is


$$\begin{array}{l} \underset{\pi }{\min}\;{CV\text{aR}}_{\alpha }\left(z\right)=\underset{\pi ,\eta }{\min}\eta +\frac{1}{1-\alpha }E\left[{\left(Z-\eta \right)}_{+}\right], \end{array}$$ (9)

subject to chance-constrained safety (Section 3.4) and integrator dynamics, where (.)_+_ = max(., 0 ).

In Equation 9, η∈ℝ is an auxiliary decision variable corresponding to the Value-at-Risk (VaR) level for the cumulative cost *Z*; the term *E*[(*Z*−η)_+_]/(1−α) then yields the standard CVaR representation as the expected excess cost in the worst (1−α) tail.

The constrained MDP (CMDP) form is


$$\begin{array}{l} \underset{\pi }{\min}𝔼\left[\overset{T-1}{\underset{t=0}{\sum }}c\left(.\right)\right]\text{s.t. }\left[\overset{T-1}{\underset{t=0}{\sum }}I\left\{g_{j}\left(.\right)>0\right\}\right]\leq \kappa_{j},\forall_{j}. \end{array}$$ (10)

Here *I*{·} denotes the indicator function, i.e., *I*{*A*} = 1 if the predicate *A* is true and 0 otherwise. The constants κ_*j*_≥0 are per-episode safety budgets that upper bound the expected number of timesteps in which constraint *g*_*j*_(·) is violated (e.g., wall or human-safety violations), thereby defining the feasible policy set in the CMDP. A Lagrangian relaxation yields multipliers λ_*j*_≥0 and the penalized objective $\underset{t}{\sum }\left[c+\underset{j}{\sum }\lambda_{j}I\left\{g_{j}>0\;\right\}\right].$

*Notation and risk functional*. Let ρ_α_(·) denote the CVaR at confidence level α∈(0, 1), defined as the expected cost in the worst 1−α tail of the distribution, so that ρ_α_(*J*) is the tail expectation of the episodic cost *J* over trajectories whose cost lies in the worst (1−α) quantile of the distribution. To avoid symbol overloading, we distinguish between the CMDP discount factor γ_*disc*_∈(0, 1), used in the value function and policy optimization, and the discrete-time CBF decay parameter γ_*cbf*_∈(0, 1), which governs how fast the safety function is allowed to decrease along closed-loop trajectories. The dual variables λ_*k*_ associated with each safety constraint are updated by projected gradient ascent and clipped to [0, λ_max_], where λ_max_>0 bounds the influence of constraint costs. The mapping Φ(·) denotes the feature embedding that converts raw geometric, forecast-derived, and latency features into the fixed-dimensional augmented state *s*_*t*_ observed by the Safe-RL policy.

### 3.5 Forecast-driven risk features and chance constraints

A transformer-based predictor provides multi-step, multi-modal distributions for each pedestrian:


$$\begin{array}{l} Y_{t+\tau }^{i}\sim p_{t+\tau }^{i}\left(.|H_{t}\right),\;\tau =1:H, \end{array}$$ (11)

where $H_{t}$ collects recent trajectories, map context, and occlusion masks. For tractability, we assume Gaussian mixture models (GMMs) or samples.

#### 3.5.1 Occupancy risk field

Define a continuous occupancy intensity for horizon τ:


$$\begin{array}{l} \Phi_{t}\left(z,\tau \right)=\overset{N_{t}}{\underset{i=1}{\sum }}\overset{K_{i}}{\underset{\kappa =1}{\sum }}\pi_{i\kappa }.N\left(z;\mu_{i\kappa }\left(\tau \right),{\sum \left(\tau \right)}_{i\kappa }\right), \end{array}$$ (12)

with mixture weights π_*ik*._ The following risk features are fed to the policy:


$$\begin{array}{l} \varrho_{t}=[\underset{\text{local occupancy}}{\underset{︸}{\max_{\tau \leq H}\Phi_{t}(p_{t},\tau )}},\underset{\text{near-field mass}}{\underset{︸}{\int \max_{\tau \leq H}\Phi_{t}(z,\tau )dz_{B(p_{t},\rho )}}},\\ \;\underset{\text{clearance forecast}}{\underset{︸}{\min_{\tau \leq H}\min_{i}E||p_{t}-Y_{t+\tau }^{i}||}}] \end{array}$$ (13)

#### 3.5.2 Chance-constrained safety with respect to predicted humans

For each *i*, τ impose


$$\begin{array}{l} \mathbb{P}\left(||p_{t}-Y_{t+\tau }^{i}|{|}_{2}\geq R_{\text{safe}}\right)\geq 1-\epsilon . \end{array}$$ (14)

If $Y_{t+\tau }^{i}\;\sim N\left(\mu ,\Sigma \right),$ a conservative Gaussian chance-constraint via the one-sided Chebyshev/ellipsoidal bound gives


$$\begin{array}{l} h_{i,\tau }\left(p_{t}\right):=\underset{\text{mean clearance}}{\underset{︸}{||p_{t}-\mu |{|}_{2}}}-\;\underset{\text{uncertainty margin}}{\underset{︸}{\kappa_{1-\epsilon }\sqrt{\lambda_{max}\left(\Sigma \right)}}}-R_{\text{safe}}\geq 0, \end{array}$$ (15)

with $\kappa_{1-\epsilon }=\Phi^{-1}\left(1-\epsilon \right)$. Equivalently, in squared form for CBF design:


$$\begin{array}{l} {\tilde{h}}_{i,\tau }\left(p_{t}\right):={\left(||p_{t}-\mu |{|}_{2}-\kappa_{1-\epsilon }\sqrt{\lambda_{\max}\left(\Sigma \right)}\right)}^{2}-R_{\text{safe}}^{2}\geq 0. \end{array}$$ (16)

The dynamic safety set becomes


$$\begin{array}{l} S_{\text{dyn}}\left(t\right)=\underset{i,\tau }{⋂}\left\{x:{\tilde{h}}_{i,\tau }\left(p_{t}\right)\geq 0\right\}. \end{array}$$ (17)

The overall safe set is $S\left(t\right)=S_{\text{stat}}\cap S_{\text{dyn}}\left(t\;\right).$

### 3.6 Discrete-time CBF constraints

For each safety function *h*(*x, t*) (static walls; predicted humans), discrete-time forward invariance is enforced by the inequality


$$\begin{array}{l} h\left(x_{t+1},t+\Delta t\right)-\left(1-\gamma \right)h\left(x,t\geq 0,\gamma \in \left(0.1\right)\;\right] \end{array}$$ (18)

which guarantees *h*≥0⇒*h* remains non-decreasing up to decay γ.

Using first-order dynamics linearization at (*x*_*t*_, *u*_*t*_):


$$\begin{array}{l} h\left(x_{t+1}\right)\approx h\left(x_{t}\right)+\nabla_{x}{h\left(x_{t}\right)}^{⊤}\left(x_{t+1}-x_{t}\right)\\ \;=h\left(x_{t}\right){+}_{t}\nabla_{x}{h\left(x_{t}\right)}^{⊤}f\left(x_{t},u_{t}\right). \end{array}$$ (19)

Thus, a linear constraint in *u*_*t*_:


$$\begin{array}{l} \underset{A_{h}\left(x_{t}\right)}{\underset{︸}{-\Delta_{t}\nabla_{x}h{\left(x_{t}\right)}^{⊤}\frac{\partial f}{\partial u}\left(x_{t}\right)u_{t}}}\\ \leq \underset{b_{h}\left(x_{t}\right)}{\underset{︸}{h\left(x_{t}\right)-\Delta_{t}\nabla_{x}h{\left(x_{t}\right)}^{⊤}f\left(x_{t},0\right)-\left(1-\gamma \right)h\left(x_{t}\right)}} \end{array}$$ (20)

- Static wall CBF: $h_{\text{wall}}\left(x\right)=d_{O}\left(p\right)-R_{\text{wall},}$

$$\begin{array}{l} \nabla_{x}h={\left[\nabla_{p}d_{O}^{⊤},0,0\right]}^{⊤}. \end{array}$$ (21)

- Pedestrian CBF (chance-robust): use ${\tilde{h}}_{i,\tau }\left(x\right)$ with $\nabla_{p}\tilde{h}=2\left(||p-\mu |{|}_{2}-\delta \right)\frac{\left(p-\mu \right)}{||p-\mu |{|}_{2}}$, where $\delta =\kappa_{1-\epsilon }\sqrt{\lambda_{\max}\left(\Sigma \;\right)}.$

Collecting all active CBFs yields


$$\begin{array}{l} A_{t}u_{t}\leq b_{t},\;A_{t}=\left[\begin{array}{c} A_{h_{1}}\\ \vdots \\ A_{h_{m}} \end{array}\right],b_{t}=\left[\begin{array}{c} b_{h_{1}}\\ \vdots \\ b_{h_{m}} \end{array}\right]. \end{array}$$ (22)

### 3.7 Latency-aware state prediction

Let Δ_sens_ be sensing-to-actuation latency and Δ_net_ a teleoperation network delay. The control acts on *x*_*t*+Δ_, Δ = Δ_sens_+Δ_net_. A predictive state is used:


$$\begin{array}{l} {\hat{x}}_{t+\Delta }=x_{t}+\Delta f\left(x_{t},u_{t-1}\right),\;{\hat{p}}_{t+\Delta }=p_{t}\Delta +v_{t}{\left[\cos\theta_{t},\sin\theta_{t}\right]}^{⊤}. \end{array}$$ (23)

All CBF and chance constraints are evaluated at ${\hat{x}}_{t+\Delta }$ to pre-empt delay effects.

### 3.8 Shielded action via quadratic program

Given a nominal policy action from the Safe-RL controller, the shield solves


$$\begin{array}{l} u_{t}^{⋆}=\;\arg\underset{u\varepsilon {\mathbb{R}}^{2}}{\min}||u-u_{t}^{\text{nom}}|{|}_{2}^{2} \end{array}$$ (24)

$$\begin{array}{l} A_{t}\left({\hat{x}}_{t+\Delta }\right)u\leq b_{t}\left({\hat{x}}_{t+\Delta }\right), \end{array}$$ (25)

$$\begin{array}{l} u_{\min}\leq u\leq u_{\max}. \end{array}$$ (26)

The solution minimally perturbs the nominal action while guaranteeing discrete-time CBF invariance under uncertainty margins. With *m* active constraints and a 2D control, the computational complexity is *O*(*m*) for active-set QPs.

### 3.9 Augmented MDP state for learning

The Safe-RL policy observes an augmented state.


$$\begin{array}{l} s_{t}=\left[x_{t},p_{\text{goal}}-p_{t},\;\underset{\text{map SDF features}}{\underset{︸}{\text{Ψ}\left(p_{t}\right)}},\;\underset{\text{forecast risk}}{\underset{︸}{\varrho_{t}}},\;\underset{\text{latency features}}{\underset{︸}{\xi_{t}}}\right], \end{array}$$ (27)

where $\psi (p_{t})=[d_{O}(p_{t}),\;\nabla_{p}d_{O}{\left(p_{t}\right)}^{⊤}]\;$and ξ_*t*_ = [Δ_sens_, Δ_net_], and the action space is $U=u:u_{\min}\leq u\leq u_{\max}$.

### 3.10 Assumptions and feasibility

1. The map SDF $d_{O}$ is Lipschitz and differentiable almost everywhere; $||\nabla_{p}d_{O}||\leq \;1$.
2. Forecast covariance ∑ admits λ_*max*_(∑) and is bounded on [*t, t*+*H* ].
3. Control bounds ensure QP feasibility under mild backup policies; if infeasible, a fallback braking *u* = [*a*_*min*_, 0] is admissible and yields a non-decreasing function for static walls.

A transformer supplies predictive distributions; risk features and chance constraints encode uncertainty; a Safe-RL policy proposes actions; and a discrete-time CBF-QP shield enforces invariance under latency and occlusion. This mathematical scaffold supports the subsequent algorithmic and experimental components.

## 4 Methodology

The proposed study describes a detailed end-to-end forecast → policy → safety-filter framework that enables anticipatory, safety-assured co-navigation for a TPR in hospital layouts. The method comprises three tightly coupled layers: (i) a transformer-based human-motion forecaster that outputs multi-step, multi-modal trajectory distributions; (ii) a Safe-RL controller that consumes risk features derived from the forecasts and optimizes a constrained objective; and (iii) a discrete-time CBF shield that projects the controller's actions into a provably safe set at run time, with latency compensation. Operationally, the transformer's predicted multi-agent distributions constitute an implicit belief state, summarizing the Bayes-filter update and entering the Safe-RL policy via risk-aware occupancy features, while the CBF shield enforces hard safety on the resulting actions. Implementation choices are reported to ensure full reproducibility and to support ablation studies. Table 2 summarizes the run-time CBF shield and latency-compensation design, detailing safety functions, decay factor, QP formulation, slack handling, solver choice, and diagnostic metrics.

**Table 2:** CBF shield and latency compensation.

**Element**	**Setting/value**
Safety functions	Wall CBF; human chance-robust CBF
Decay γ	0.1–0.3
QP	min|*u*−*u*^*nom*^|^2^ s.t. *A u* ≤ *b*, bounds
Slack	Non-neg.; penalty 10^3^-10^4^
Latency Δ	Sensing + network; predictive state ${\hat{x}}_{t+\Delta }$
Solver	Active-set or QP-OASES-like; <1 ms typical
Diagnostics	Residuals; slack count; constraint actives

### 4.1 Transformer-based human-motion forecaster

This subsection describes the transformer-based human-motion forecaster that supplies short-horizon, uncertainty-aware multi-agent trajectory distributions used as risk features for downstream control.

#### 4.1.1 Inputs

At each time step *t*, the forecaster receives a sliding window of tracked pedestrian states ${\left\{y_{t-l:t}^{i}\right\}}_{i=1}^{N_{t}}$ (2-D positions with optional velocities), the robot pose *x*_*t*_, a local map patch (SDF and visibility mask), and agent-centric features (pairwise displacements and occupancy raster). Missing detections due to occlusion are explicitly masked.

#### 4.1.2 Architecture

**Figure 2:**
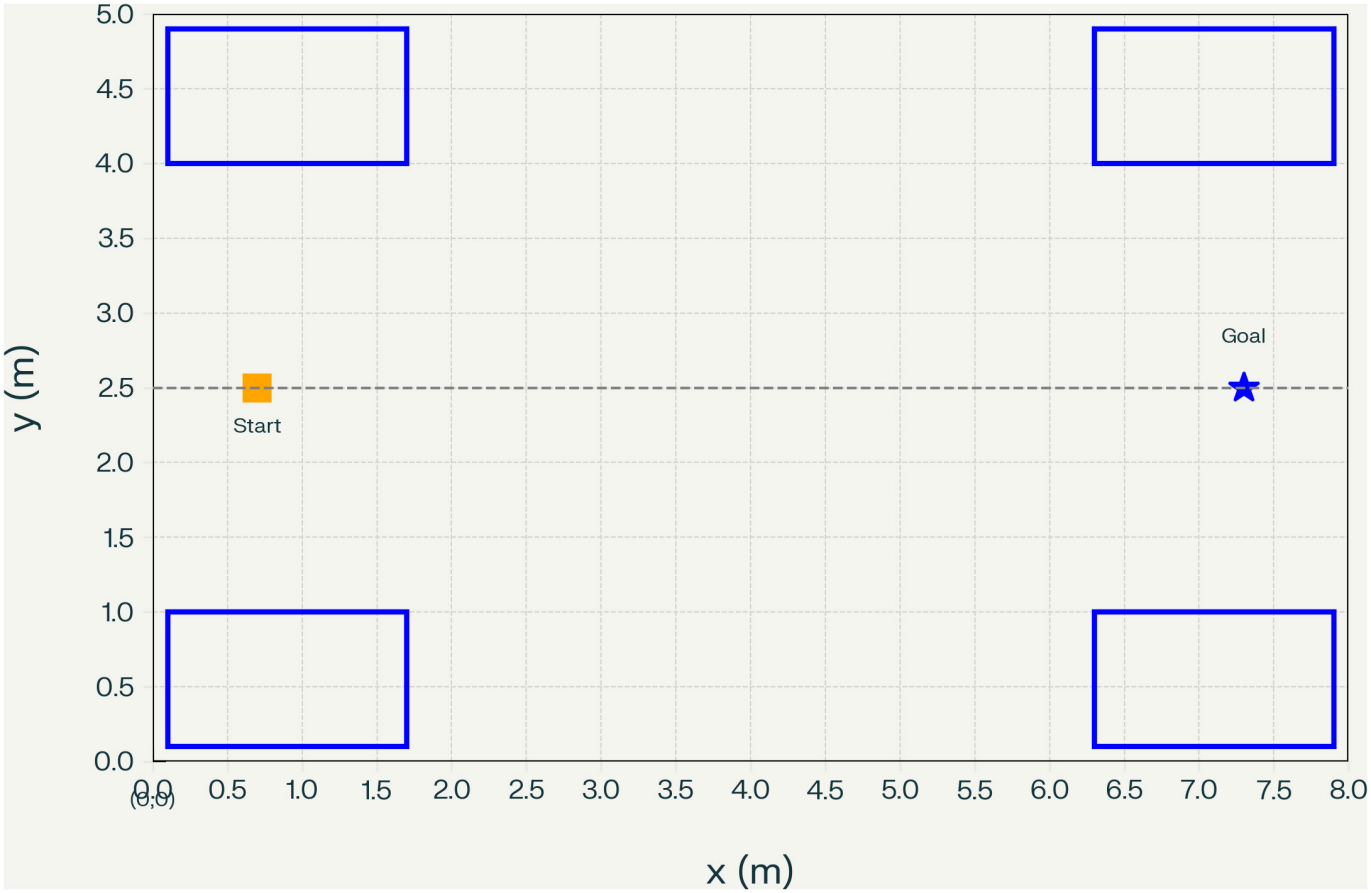
Experimental environment: top–down ward floor plan used in simulation. Walls, doors, beds, curtains, and equipment define the navigation space for the telepresence robot and simulated pedestrians; start and goal regions are indicated schematically.

A light, latency-aware transformer is used in the system architecture of the proposed solution, as shown in Figure 2:

- Tokenization: per-agent temporal tokens and contextual map tokens.
- Encoder: multi-head self-attention over agent tokens to capture social interactions.
- Cross-attention: agent tokens attend to map tokens (doors, walls, bottlenecks).
- Decoder: non-autoregressive, predicting *H* steps for each agent.
- Output head: per-step Gaussian mixture parameters {π_*ik*_, μ_*ik*_, Σ_*ik*_}.

The transformer forecaster is configured with shallow depth and a limited number of heads and tokens to satisfy real-time inference requirements on telepresence platforms; all architectural components above remain unchanged and are executed at each control cycle.

#### 4.1.3 Uncertainty calibration

Temperature scaling is applied to mixture variances, and a quantile-matched scaling on Mahalanobis distances is used to align predicted covariances with empirical errors (calibration set only). To reduce overconfidence, each Gaussian component is constrained by a variance floor to prevent eigenvalues from collapsing to unrealistically small values, and calibrated Mahalanobis distances are matched to empirical error quantiles on a held-out set. These calibrated covariances feed directly into the chance-robust CBF design (Equations 14–17), which uses a high-quantile safety factor to convert forecast uncertainty into conservative clearance margins. Probabilistic calibration is further assessed in Section 6 via reliability diagrams of predicted near-miss risk vs. empirical frequency, where the proposed method tracks the diagonal more closely than PPO and DWA.

#### 4.1.4 Training objective

Negative log-likelihood (NLL) over future trajectories with an uncertainty regularizer:


$$\begin{array}{l} L_{pred}=-\underset{i,t,\tau }{\sum }\log\left(\underset{k}{\sum }\pi_{ik}N\left(y_{t+\tau }^{i}|\mu_{ik}\left(\tau \right),\Sigma_{ik}\left(\tau \right)\right)\right)\\ \;+\lambda_{\Sigma }\underset{i,t,\tau }{\sum }\text{tr}\left(\Sigma_{ik}\left(\tau \right)\right) \end{array}$$ (28)

Table 3 consolidates the transformer forecaster specification—inputs, tokenization, architecture, horizon, uncertainty calibration, and training hyperparameters—serving as the canonical configuration for all forecasting experiments.

**Table 3:** Transformer forecaster: model specification and training hyperparameters.

**Component**	**Setting/value**
History window ℓ	8–12 steps
Horizon *H*	8–16 steps
Tokens	Agent time tokens; map tokens (SDF + visibility)
Encoder	2–4 layers; 4–8 heads
Decoder	Non-autoregressive; per-step heads
Output	GMM per agent per step (*K* = 3–5)
Loss	NLL + variance regularizer
Calibration	Temperature scaling; quantile matching
Occlusion	Binary masks on tokens; covariance inflation
Optimizer	Adam; lr 1*e*−4 *to* 3*e*−4
Batch	64–128 sequences

#### 4.1.5 Risk features for control

The predicted distributions are converted into compact features ϱ_*t*_: (i) maximum occupancy intensity around the robot, (ii) near-field probability mass within a radius ρ, (iii) forecasted minimum clearance; and (iv) a flow-direction histogram (optional) to disambiguate counter-flows.

### 4.2 Safe reinforcement-learning controller

This subsection details the Safe-RL controller, formulated as a constrained Markov decision process that consumes forecast-derived risk features to optimize task performance within safety budgets.

#### 4.2.1 CMDP setup

The controller solves a constrained MDP with a risk-sensitive objective and constraint budgets on safety violations. This module instantiates the Safe-RL formulation of the CMDP in Section 3.3, where safety is expressed as expected counts of CBF-slack activations and near-miss events, and risk sensitivity is captured via a CVaR-style auxiliary objective. The augmented observation is:


$$s_{t}=[x_{t},p_{\text{goal}}-p_{t},\psi (p_{t}),\varrho_{t},\xi_{t}],$$ (29)

Here, *s*_*t*_ concatenates the robot state *x*_*t*_, the forecaster-derived risk features *r*_*t*_, static SDF-based map features ϕ_*SDF*_(distances to walls, doors, and bottlenecks), and a scalar Δencoding sensing-and-actuation latency. This augmented observation exposes both anticipatory risk information and latency-aware geometry to the Safe-RL controller.

#### 4.2.2 Policy and value function

Two MLPs (actor and critic) are used. The actor outputs a Gaussian distribution over [*a*_*t*_, ω_*t*_] with state-dependent mean and diagonal covariance; squashing enforces action bounds.

#### 4.2.3 Learning algorithm

A Lagrangian PPO variant is used:

- Primary objective: expected return with CVaR surrogate (an auxiliary head estimates tail risk).
- Constraints: expected counts of CBF violations (from shield diagnostics) and near-miss events (≤ 0.3 *m* ).
- Dual updates: per-constraint multipliers updated by projected gradient ascent.
- Exploration: off-policy replay is not used; entropy regularization stabilizes exploration.

In effect, the actor parameters θ and critic parameters are updated to maximize the clipped PPO surrogate while keeping empirical estimates of the constraint costs (shield slack activations and near-miss events) below their budgets, with Lagrange multipliers adapting as penalty weights whenever these costs exceed the specified thresholds.

In principle, one could differentiate through the CBF-QP and treat the shield as part of the policy, using implicit-function gradients so that barrier parameters directly shape the actor update. We deliberately keep the shield modular and non-differentiated: the CBF parameters are tuned at the control layer to preserve clear forward-invariance guarantees and to allow conservative, certifiable fallbacks even when the policy is updated. Exploring differentiable CBF shields and tighter end-to-end coupling between the barrier and the policy is left as future work.

#### 4.2.4 Rewards and costs (shaped)

- Progress reward *r*_prog_ = η(∥*p*_*t*−1_−*p*_*g*_∥−∥*p*_*t*_−*p*_*g*_∥).
- Smoothness penalty on |*a*_*t*_| and |ω_*t*_ |.
- Social-comfort cost proportional to $\underset{i}{\sum }\phi \left(∥p_{t}-y_{t}^{i}∥\;\right)$.
- Terminal success bonus; collision termination with a large penalty.

Table 4 enumerates the CMDP design and optimization details for the Safe-RL controller, including state composition, constraints, reward/cost shaping, and learning settings used across evaluations.

**Table 4:** Safe-RL controller: CMDP design and optimization.

**Item**	**Setting/value**
State*s*_*t*_	[[*x*_*t*_, *p*_*g*_−*p*_*t*_, ψ(*p*_*t*_), ϱ_*t*_, ξ_*t*_]]
Action *u*_*t*_	[*a*_*t*_, ω_*t*_], bounded
Algorithm	PPO–Lagrangian (on-policy)
Risk	CVaR head (tail quantile)
Constraints	CBF slack rate; near-miss rate; wall proximity
Rewards	Progress; smoothness; terminal bonus
Penalties	Collision; shield slack; jerk (optional)
Entropy	0.001–0.01
Discount γ	0.99
GAE λ	0.95
Update	3–10 epochs per batch

#### 4.2.5 Design choices and technical rationale

The technical design of the framework was guided by the need to balance anticipatory perception quality, formal safety guarantees, and real-time deployability on embedded TPR hardware. The transformer-based forecaster was selected over recurrent or graph-based alternatives because short-horizon self-attention captures inter-agent interactions while admitting parallel inference and fixed-latency decoding; the non-autoregressive output head further mitigates error accumulation across the 0.8–1.6 s prediction horizon. The occupancy-field risk representation compresses multi-agent trajectory distributions into low-dimensional features that remain stable under occlusions and are directly usable for constructing chance-robust CBF constraints.

On the control side, navigation was formulated as a constrained Markov decision process with an auxiliary CVaR objective, instead of a purely expected-return PPO formulation, to shape the policy toward tail-risk-averse behavior in dense crowds and rare but critical events. The CBF-based safety shield is kept modular and model-based to retain forward-invariance guarantees even when the learned policy encounters out-of-distribution states; slack variables and shield diagnostics are tuned to prioritize feasibility while exposing interpretable intervention statistics during training. Finally, the forecast horizon, state augmentation, and reward/cost shaping (Table 4) were empirically calibrated to trade off efficiency against social comfort: shorter horizons degraded anticipation of crossing pedestrians, whereas longer horizons increased forecast drift and CBF conservatism, reducing success rates and inducing stop-and-go behavior. To prevent “shield myopia,” the critic receives the shield's dual residuals and action deviations as inputs; the actor is regularized toward low-intervention regimes.

#### 4.2.6 Safety during learning

The shield runs online during training to avoid unsafe data collection. Let $a_{t}^{\pi }$ denote the unconstrained action sampled from the policy and $a_{t}^{\text{sh}}$ denote the shielded action returned by the QP, and define the intervention vector $\delta a_{t}=a_{t}^{\text{sh}}-a_{t}^{\pi }$. Let λ_*t*_ denote the vector of optimal dual variables (Lagrange multipliers) of the CBF constraints in the QP.

During actor–critic updates, trajectories are rolled out using the shielded controls $a_{t}^{sh}$, and returns and constraint costs are always computed under these shielded dynamics. Policy gradients use $\log\pi_{\theta }\left(a_{t}^{\pi }|s_{t}\right)$ together with advantages Â_*t*_ estimated from the shielded trajectories, so that the critic learns values conditioned on (*s*_*t*_, δ*a*_*t*_, λ_*t*_) and the policy is nudged toward regions of the action space where the shield intervenes rarely and weakly. This design does not eliminate “myopia” entirely but empirically reduces it by making shield activity explicitly visible to both the value function and the regularized actor.

### 4.3 Discrete-time CBF safety shield

This subsection presents the discrete-time CBF safety shield, which wraps the Safe-RL policy in a quadratic program that enforces forward invariance of safety sets under forecast uncertainty and latency.

#### 4.3.1 Safety functions

Two families are enforced at each step: (i) wall CBFs using the map SDF; (ii) human CBFs that incorporate forecast uncertainty (chance-robust function $\tilde{h_{i,\tau }}$).

#### 4.3.2 Inequalities

For each active safety function *h*, the discrete-time CBF condition:


$$\begin{array}{l} h\left(x_{t+1}\right)-\left(1-\gamma \right)h\left(x_{t}\right)\geq 0 \end{array}$$ (30)

is linearized to an affine constraint in the controls:


$$\begin{array}{l} A_{h}\left(x_{t}\right)u_{t}\leq b_{h}\left(x_{t}\right). \end{array}$$ (31)

All active constraints are stacked into *A*_*t*_*u*_*t*_ ≤ *b*_*t*_.

#### 4.3.3 QP projection

The shield solves


$$\begin{array}{l} u_{t}^{⋆}=\arg\underset{u}{\min}∥u-u_{t}^{nom}{∥}_{2}^{2}\;s.t.A_{t}u\leq b_{t},\\ {\;u}_{\min}\leq u\leq u_{\max} \end{array}$$ (32)

The QP returns both the shielded control $a_{t}^{sh}$ and the vector of Lagrange multipliers $\lambda_{t}\in {\mathbb{R}}_{\geq 0}^{m}$ associated with the active linearized CBF constraints. The entries of λ_*t*_ quantify how tight each safety constraint is at the optimum, and, together with the slack variables, they provide a compact diagnostic signal describing the strength and frequency of shield interventions. A small slack with a large penalty is allowed for numerical feasibility; slack activations are logged as violations for the CMDP constraints.

All CBFs are evaluated at a predictive state ${\hat{x}}_{t+\Delta }$ to counter sensing and network delays. The same prediction feeds the QP.

### 4.4 End-to-end control cycle

The proposed co-navigation stack executes at 30–50 Hz and implements a forecast → policy → safety-filter pipeline with explicit latency compensation. At each control tick, detections are tracked, short-horizon human-motion distributions are predicted, compact risk features are constructed, and an augmented state is formed. A Safe-RL actor emits a nominal action that is projected by a discrete-time CBF-QP shield evaluated at a latency-compensated predictive state. The shielded action is applied, diagnostics are logged, and (in training mode) policy and dual variables are updated. The full loop is summarized in Algorithm 1.

Algorithm 1 End-to-end forecast → policy → safety-filter control loop. 
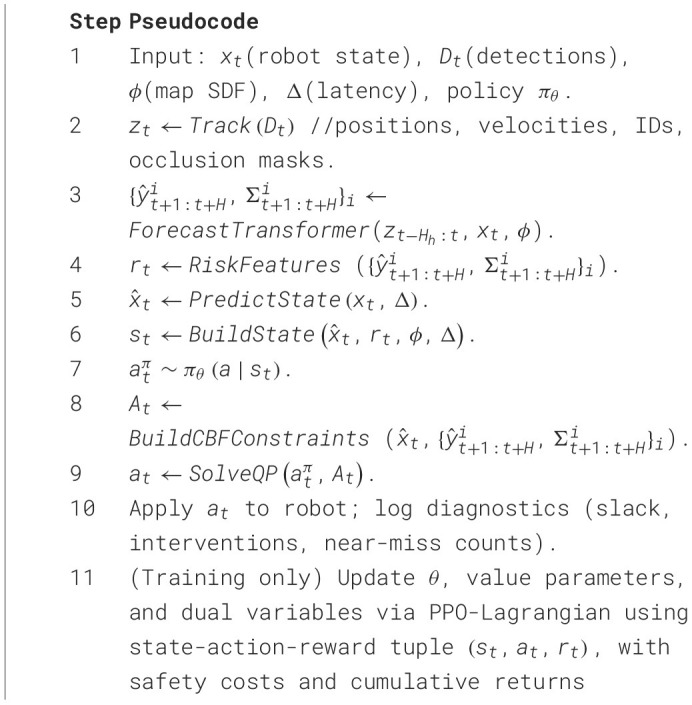

*ForecastTransformer* returns multi-step, multi-modal pedestrian state distributions. *RiskFeatures* compacts these into occupancy and clearance summaries. *BuildCBFConstraints* assembles wall and chance-robust human CBFs evaluated at *x*_*t*+Δ_. *SolveQP* is a 2-variate active-set QP with optional slack (large penalty) to ensure numerical feasibility in rare edge cases.

Pipeline: Sensing and tracking → detection, data association, occlusion masks. Forecasting → transformer predicts *H* steps for visible and recently visible agents. Risk features → compute ϱ_*t*_ from the predicted distributions. Policy → actor returns $u_{t}^{nom}$ given *s*_*t*_. Shield → evaluate CBFs at ${\hat{x}}_{t+\Delta }$ and solve the QP for $u_{t}^{⋆}$. Execution → send $u_{t}^{⋆}$ to the robot; log shield residuals and slack. Learning (train mode) → collect transitions; update actor/critic and Lagrange multipliers in mini-batches.

### 4.5 Theoretical properties (sketch)

This subsection explains how sensing and network delays are modeled using a latency-aware predictive state, which is consistently used in both the CBF constraints and the shield's QP to pre-empt delay-induced violations.

#### 4.5.1 Forward invariance

If for all active safety functions *h* the linearized discrete-time CBF constraints hold, then the safe set S(t)={x:h(x,t)≥0∀h} is forward-invariant under the closed-loop dynamics with ZOH. Chance robustness enters through the uncertainty margin in $\tilde{h_{i,\tau }}$; the guarantee is conservative at level ϵ.

#### 4.5.2 Bounded intervention

The QP is the Euclidean projection of $u_{t}^{\text{nom}}$ onto the convex set $U\cap \left\{u:A_{t}u\leq b_{t}\right\}$. Hence, $|u_{t}^{⋆}-u_{t}^{\text{nom}}{|}_{2}$ is minimal among safe actions, limiting distortion of the nominal policy and supporting stable learning.

#### 4.5.3 Constraint satisfaction under latency

With a bounded model error for ${\hat{x}}_{t+\Delta }$ and Lipschitz *h*, feasibility margins scale linearly with Δ and the prediction error; the safety factor γ and SDF gradients determine the required deceleration envelope.

### 4.6 Computational complexity and real-time budget

Transformer scales as *O*((*Nℓ*)^2^) in attention; with sparse neighborhood attention, this becomes *O*(*Nℓk*), *k*≪*Nℓ*. With 15*N* ≤ 15 and ℓ ≤ 12, run time is typically <10 ms on an embedded GPU.

MLP inference is *O*(*P*) with *P* parameters and typically <0.2 ms on embedded CPUs.

The QP has two decision variables and *m* linear constraints; active-set methods run in *O*(*m*). With *m* ≤ 12, solve times <0.3 ms are typical.

The cycle fits within 20–30 ms on embedded platforms; the chosen design preserves 30–50 Hz control with headroom for sensing.

### 4.7 Ablation and diagnostic hooks

This subsection defines the augmented MDP state that bundles robot, map, forecasting, and latency features into a fixed-dimensional representation for the actor–critic networks.

- No-forecast: risk features ϱ_*t*_ removed; policy observes only instantaneous detections.
- No-shield: CBF projection disabled; constraint costs remain in the CMDP.
- Short-horizon: *H* reduced by 50%.
- Uncalibrated: temperature scaling disabled.
- High latency: Δ increased by two to three times.
- Risk-blind: CVaR head disabled; expected cost only.

Each ablation logs success rate, collision rate, minimum clearance, near-miss rate, time-to-goal, shield activation rate, and average QP deviation ∥*u*^⋆^−*u*^nom^ ∥.

The method operationalizes anticipation (transformers) and safety (Safe-RL + CBF) in a modular architecture with explicit latency handling and verifiable constraints. The tables provide a compact bill of materials for replication and for controlled ablations.

### 4.8 Implementation details

This subsection summarizes the complete forecast → policy → safety-filter control loop executed at run time.

#### 4.8.1 Tracking

A constant-velocity Kalman filter with gating on Mahalanobis distance is used for data association; tracks missing for ≤ *M* frames are carried with increased covariance.

#### 4.8.2 Forecast horizon

*H*∈[8, 16] steps (0.8–1.6 s at 10 Hz) balance look-ahead and drift. Mixture components per agent *K*∈{ 3, 5}.

#### 4.8.3 Normalization and masking

Inputs are agent-centered; map patches are aligned to the robot's heading. Occlusions are injected as binary masks on tokens and rasters.

#### 4.8.4 Policy architecture

Actor/critic networks use two to three hidden layers with layer normalization, Tanh activations, and state-dependent log standard deviation with a lower bound.

#### 4.8.5 Training

On-policy updates occur every *N* steps with advantage estimation using GAE; Lagrange multipliers are maintained for (i) CBF slack rate, (ii) near-miss rate, and (iii) wall proximity breaches.

## 5 Experimental setup

The proposed study modeled the evaluation environment as a hospital-style ward represented by a 2D floor plan with walls, doors, beds, curtains, and fixed equipment (Figure 2). The floor plan is rasterized into an occupancy grid and an SDF, which are used by both the motion planner and the transformer's map encoder. Pedestrian traffic is generated by simulated human agents following goal-directed trajectories with ORCA-based collision avoidance, while the telepresence robot is commanded from an entrance pose to bedside goal regions using each of the navigation stacks described below.

All experiments in this work were conducted in a physics-based simulator instantiated from the ward floorplan in Figure 2. All pedestrian agents are purely virtual and evolve according to crowd motion models (e.g., ORCA-based controllers) within this simulated environment; no trajectories, sensor streams, or other measurements are collected from real patients, staff, or visitors. Accordingly, the study should be interpreted as a simulation-based evaluation of navigation algorithms in idealized hospital-ward layouts rather than as a clinical trial or observational study on human subjects.

### 5.1 Navigation algorithms

**Figure 3:**
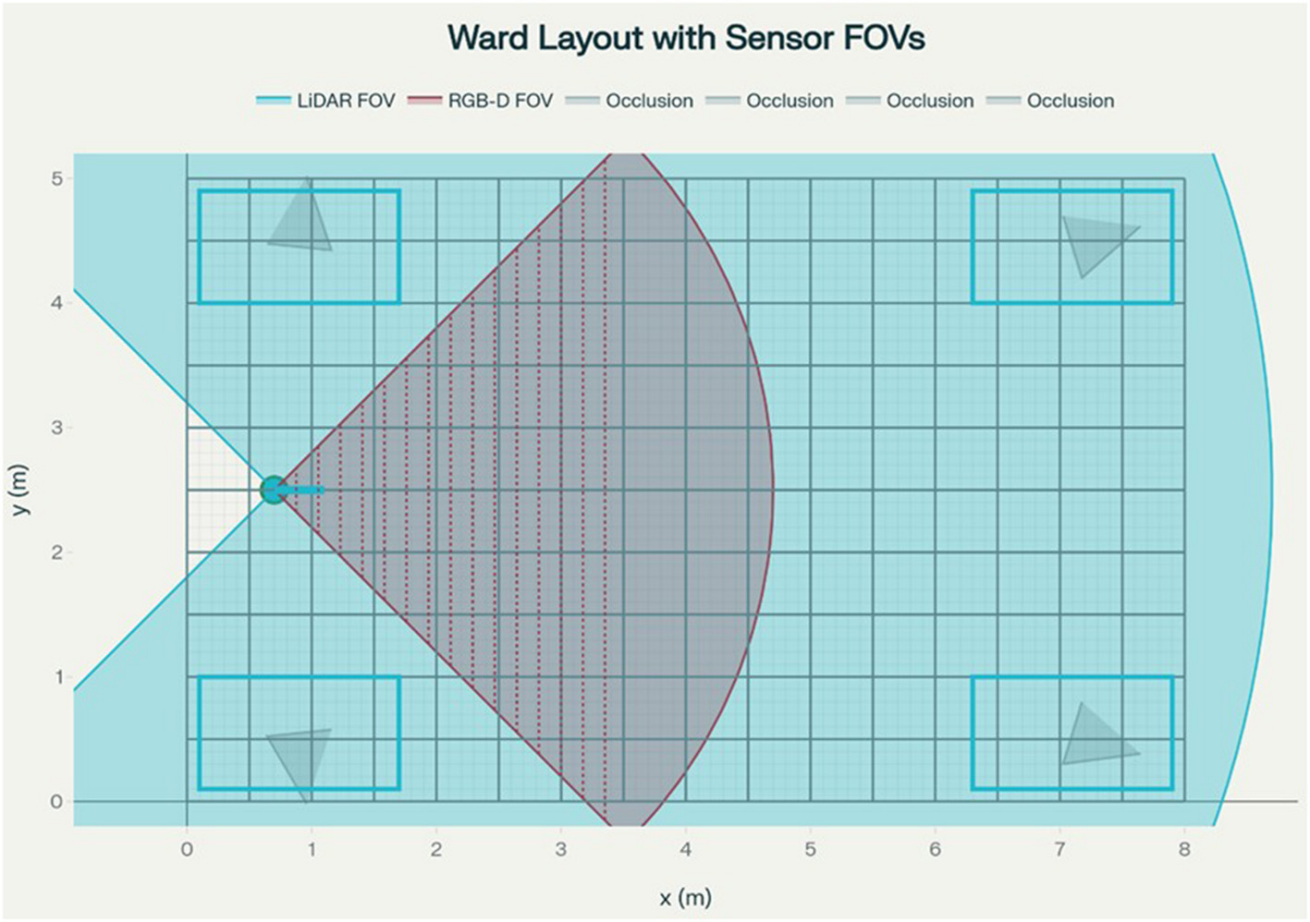
Sensor configuration.

The study compared the proposed method (transformer-based trajectory forecasting + Safe-RL planner + CBF) against standard baselines. The proposed pipeline works as follows: at each time step, a transformer neural network predicts the future positions of nearby humans (based on their past observed paths), similar to recent works such as Social-TransMotion, enabling the robot to anticipate human motion. A reinforcement-learning (RL) policy then selects a motion command; this policy is trained with safety constraints. In practice, the study implemented Safe-RL by adding a CBF-based safety layer on top of the learned policy. If the RL action violates safety constraints (e.g., approaches a human too closely), the CBF projects it to the nearest safe action. This ensures that the robot never enters an unsafe zone during training or deployment. Perception is simulated using a planar LiDAR and a forward RGB-D sensor, producing range and depth observations that populate a 0.1 m occupancy grid. Field-of-view limits and occlusions from beds and humans are explicitly modeled to mirror real line-of-sight constraints (Figure 3).

**Figure 4:**
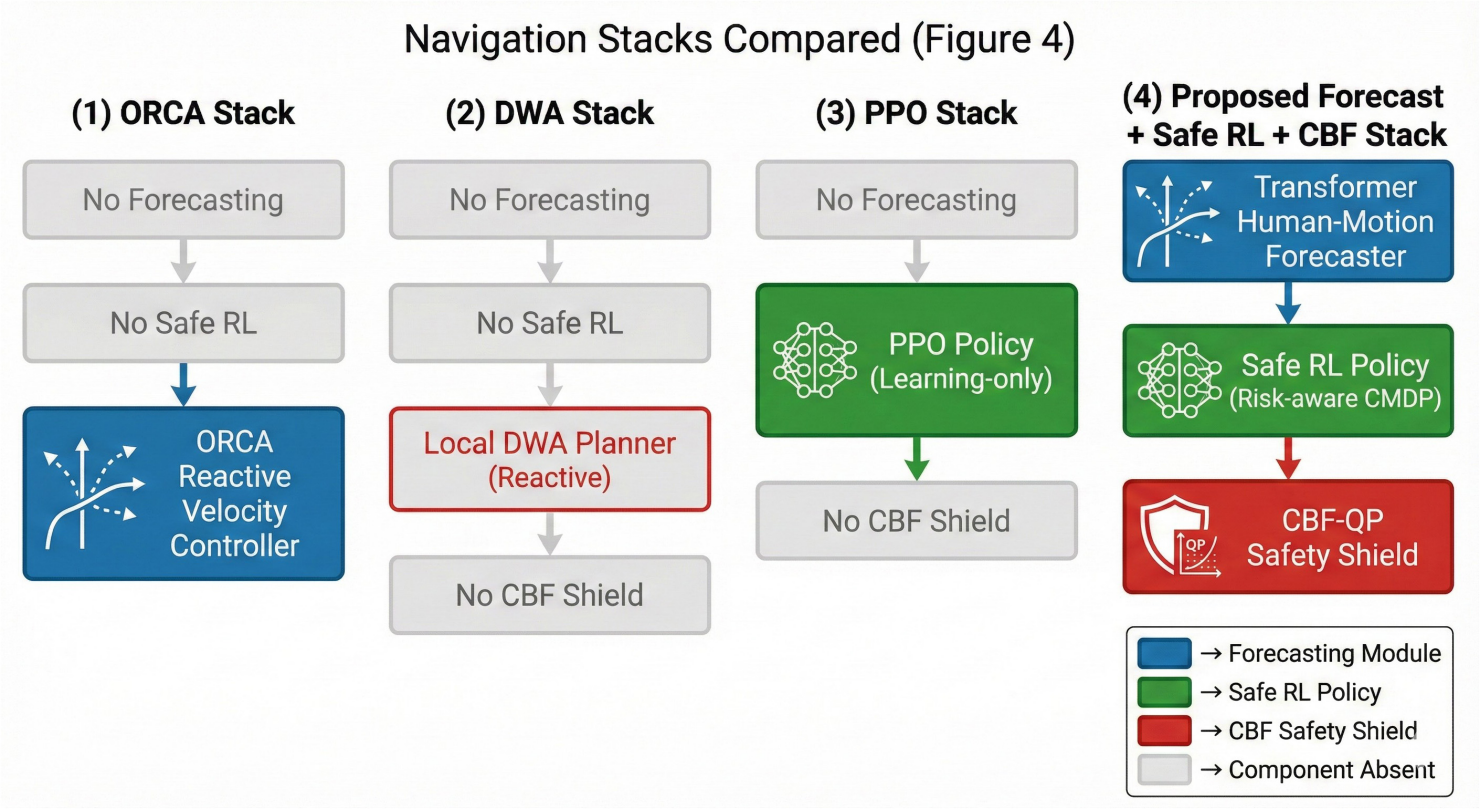
Navigation stacks compared in the experiments: ORCA (reactive multi-agent controller), DWA (classical global + local planner), PPO (learning-only controller without shield), and the proposed forecast + Safe-RL + CBF stack. Each block shows the presence or absence of forecasting, Safe-RL, and CBF safety filtering.

Baseline navigation stacks. We compare four navigation stacks, which are summarized in Figure 4:

i. ORCA stack (reactive multi-agent controller). The robot is controlled by ORCA using the RVO2 library; humans and the robot are all ORCA agents, and the robot's commanded velocity is the ORCA solution. There is no global planner, no forecasting module, and no safety shield—this stack represents a widely used reactive crowd-navigation baseline.
ii. DWA stack (classical ROS navigation). A 2D grid-based global planner (Dijkstra/A^*^) plans in the static map, while a DWA local planner selects admissible velocities using instantaneous LiDAR detections of humans as moving obstacles. This stack has no learned forecasting, no Safe-RL, and no CBF shield and represents a standard ROS-style navigation stack.
iii. PPO stack (learning-only controller). An on-policy PPO controller receives the same instantaneous observations as the proposed method but no forecast-derived risk features; it is trained with the same task reward but without the CBF safety shield. This stack isolates the effect of Safe-RL plus shielding by providing a purely learned baseline without formal safety filtering.
iv. Proposed forecast + Safe-RL + CBF stack. The full stack uses the transformer-based human-motion forecaster, risk-aware Safe-RL controller, and discrete-time CBF-QP shield described in Sections 4.1–4.3, including latency-aware state prediction and chance-robust human CBFs. All ablations in Section 6 (no-forecast, no-shield, short-horizon, risk-blind) are derived from this stack by selectively disabling components.

### 5.2 Evaluation metrics

**Figure 5:**
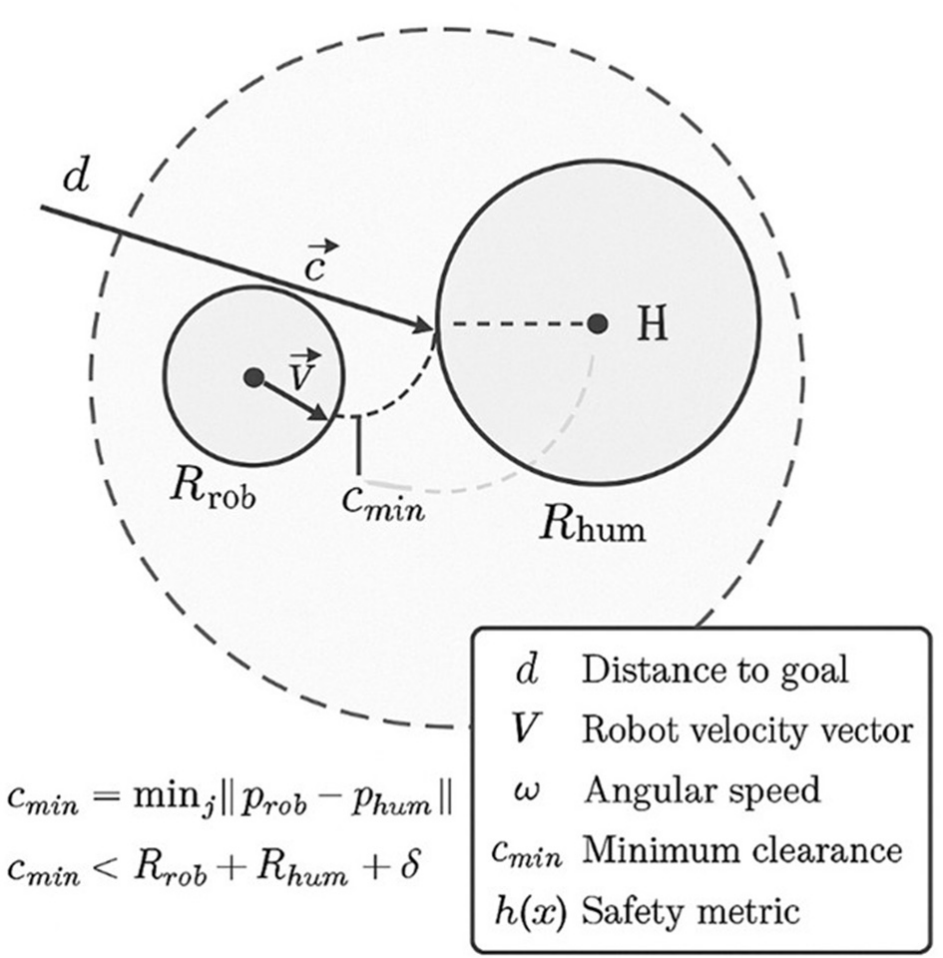
Metric geometry.

**Figure 6:**
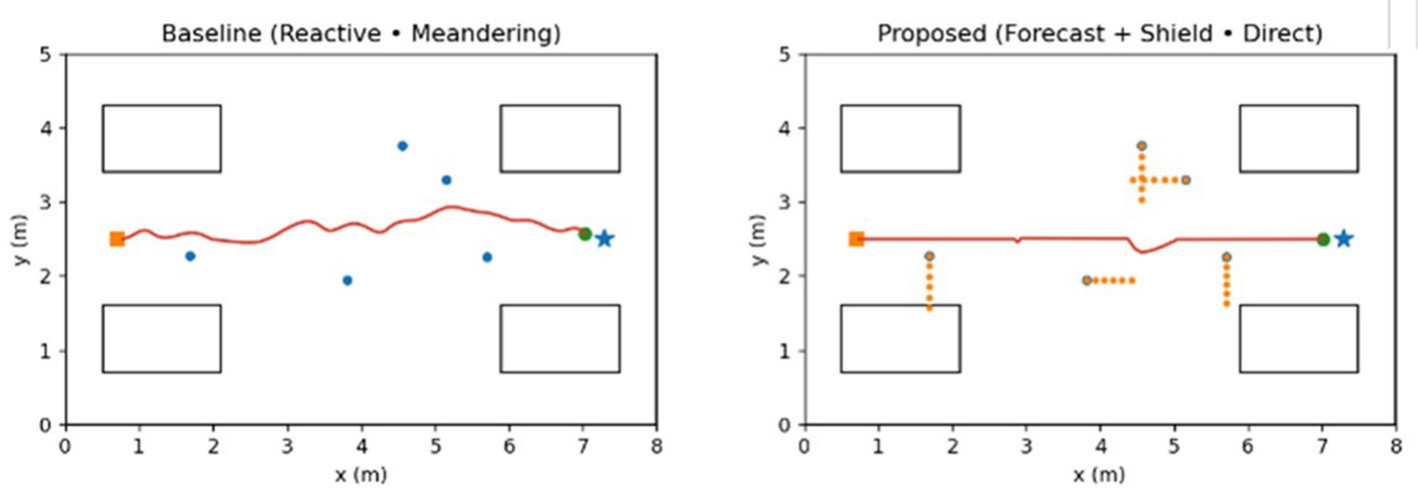
Ward scenario and representative trajectories (illustrative). **Left** Baseline reactive controller path from doorway (orange square) to doctor's station (blue star) amid pedestrians (blue dots) and beds (rectangles). **Right** Proposed forecast + CBF-shielded controller follows a near-straight route with slight evasive adjustments.

**Figure 7:**
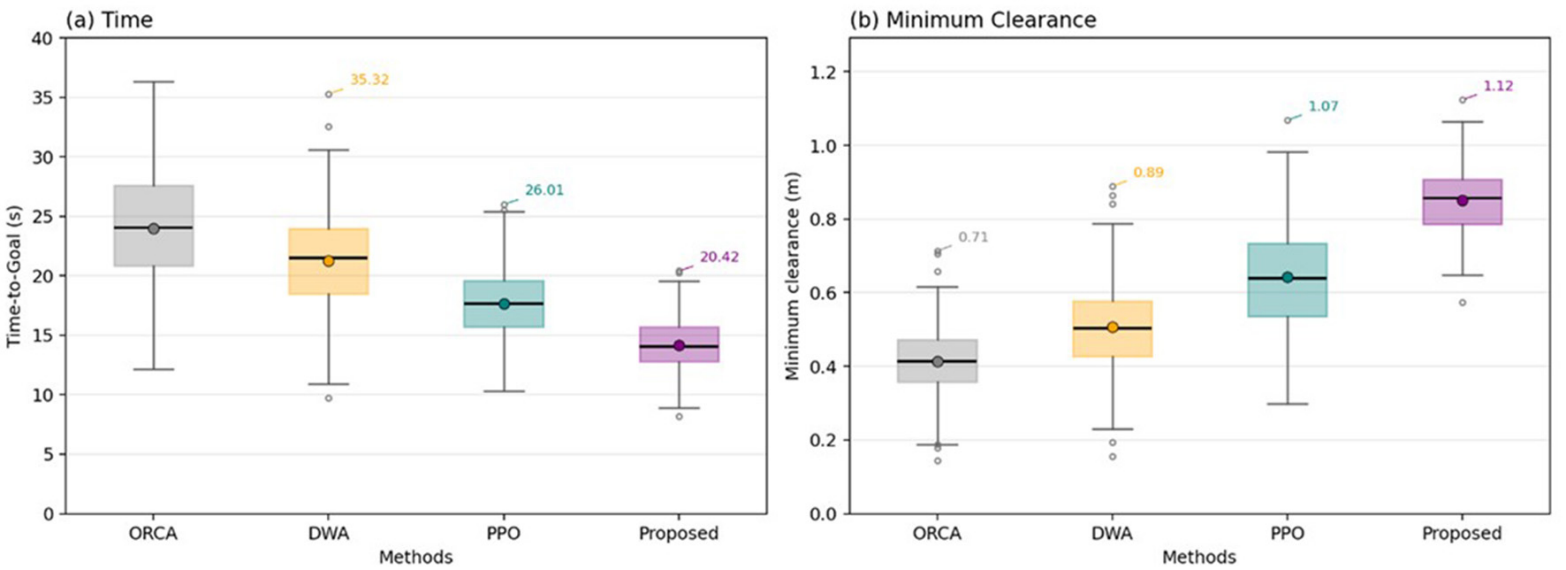
Comparison of the distributions of time-to-goal **(A)** and minimum clearance **(B)** for ORCA, DWA, PPO, and the proposed controller.

The study measured the six episode-level metrics: (i) success rate—fraction of trials in which the robot reaches the goal without any collision; (ii) collision (constraint) violations—fraction of trials with at least one collision, defined as center-to-center human–robot distance *d*_*hr*_ falling below a collision threshold *d*_coll_ = 0.2 *m*; (iii) time-to-goal—elapsed time until the robot enters the goal region or a timeout is reached; (iv) proximity violations—rate of timesteps *d*_*hr*_<*d*_prox_ = 0.5*m*, corresponding to a “personal-space” comfort radius; (v) minimum clearance—minimum *d*_*hr*_ over the episode; and (vi) near-miss rate—percentage of timesteps with 0.2*m*<*d*_*hr*_ ≤ 0.3*m*, capturing episodes in which the robot passes uncomfortably close without physical contact. Distances are computed to the nearest human or bed; path-efficiency metrics (relative path length and relative time-to-goal) are derived by normalizing against free-space runs. These definitions match the metrics reported in Tables 4–7 and Figures 5–7.

The collision threshold *d*_coll_ = 0.2*m* approximately corresponds to physical contact for the simulated TPR footprint (≈0.18*m* radius). The near-miss band (0.2 m <*d*_*hr*_ ≤ 0.3 m) and the comfort radius *d*_prox_ = 0.5*m* are aligned with proxemics studies on comfortable passing distances in human–robot encounters and with social-navigation benchmarks used in indoor environments, particularly hospital-like corridors. These values are also consistent with the comfort radius defined in the problem formulation (Section 3), and we verified that moderate variations (±0.1 m) do not alter the relative rankings of the methods.

These metrics capture human comfort: in proxemics theory, humans require a certain personal space to feel comfortable, so frequent incursions into that zone are penalized. The study also measured path length and efficiency, including relative time-to-goal and relative path length (defined as the robot's time or path length in a crowded run divided by that in a free-space run). Success rate and minimum clearance have also been used in prior work. In practice, these metrics are computed and logged in code (e.g., maintaining lists of distances and flags at each time step and aggregating post-trial). The total “incident count” (collisions + severe proximity breaches) can serve as a composite safety score. Primary outcome measures are time-to-goal, success rate, safety-margin violations, and proximity statistics; kinematic traces *V*(*t*) and ω(*t*) are also logged. Geometric definitions and the safety set enforced by the shield are summarized for reproducibility (Figure 5).

### 5.3 Visualization of simulation

As an illustrative scenario, Figure 6 shows the same ward layout from Figure 2 instantiated in the simulator, with example pedestrian trajectories and the corresponding robot paths for ORCA and for the proposed forecast-plus-shield controller, highlighting how the controller exploits anticipatory forecasting to maintain larger clearances around beds and staff. The left panel shows a reactive baseline trajectory, whereas the right panel shows the proposed forecast-plus-shield controller in the same scene.

## 6 Results

The study evaluated the proposed navigation algorithm against the baseline method using several standard performance metrics. Specifically, the measured metrics are as follows:

- Success rate—the fraction of trials in which the robot reached its goal without any collision.
- Collision (constraint) violations—the fraction of trials ending in a collision (i.e., violations of safety constraints).
- Time-to-goal (navigation time)—the time taken by the robot to reach the goal.
- Safety-margin/proximity violations—the minimum distance (safety margin) to any obstacle or human; each instance in which this distance fell below a predefined threshold was counted as a proximity violation.

The proposed method exhibits the lowest median time-to-goal with a noticeably tighter interquartile range, indicating faster and more consistent task completion while simultaneously achieving the highest median clearance and fewer low-clearance outliers. Means (filled circles) align with the medians (black bars), suggesting robustness to skew, whereas baselines show wider tails and several extreme cases. Taken together, Figure 7 shows that the proposed approach improves efficiency while preserving larger safety margins, rather than trading one for the other.

Table 5 reports the mean and standard deviation of each metric for both the baseline and proposed methods. The results clearly show that the proposed approach achieves higher efficiency and safety. For example, the proposed method achieves a mean success rate of ~98.6% (±0.4%), compared with ~96.6% (±0.8%) for the baseline, reducing the collision rate from ~3.4% to ~1.4% per trial. Similarly, the average time-to-goal is shorter for the proposed method (9.00 ± 0.3 s) than for the baseline (9.79 ± 0.5 s) (Table 2). Proximity violations are also markedly reduced under the proposed strategy. These improvements are consistent with prior work.

**Table 5:** Performance comparison between baseline and proposed navigation methods (mean ± standard deviation over all trials).

**Metric**	**Baseline (mean ±SD)**	**Proposed (mean ±SD)**
Success rate (%)	96.6 ± 0.8	98.6 ± 0.4
Time-to-goal (s)	9.79 ± 0.50	9.00 ± 0.30
Constraint violations	0.034 ± 0.010	0.014 ± 0.005
Proximity violations	0.15 ± 0.07	0.05 ± 0.02

**Figure 8:**
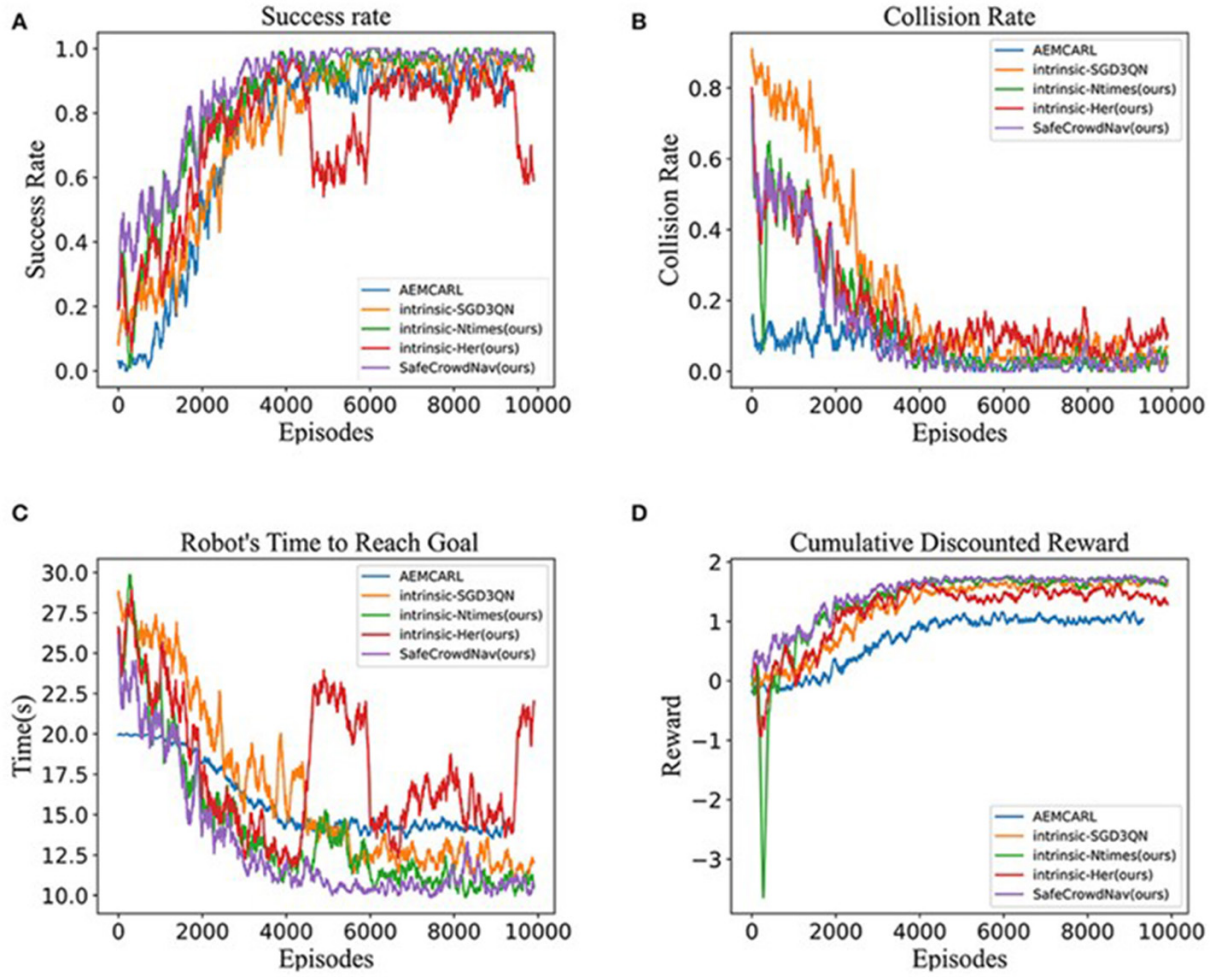
Training performance over 10,000 episodes. **(A)** Success rate, **(B)** collision rate, **(C)** time to reach goal, and **(D)** cumulative discounted reward are plotted vs. training episode.

As shown in Figure 8, the learning curves further illustrate these effects over the course of training. The success rate curve (Figure 1A) quickly rises toward 1.0 under the proposed method, whereas the baseline plateaus at a lower level. Correspondingly, the collision rate (Figure 1B) for the proposed method drops to zero much more rapidly. Time-to-goal (Figure 1C) also converges to a lower value for the proposed algorithm.

**Figure 9:**
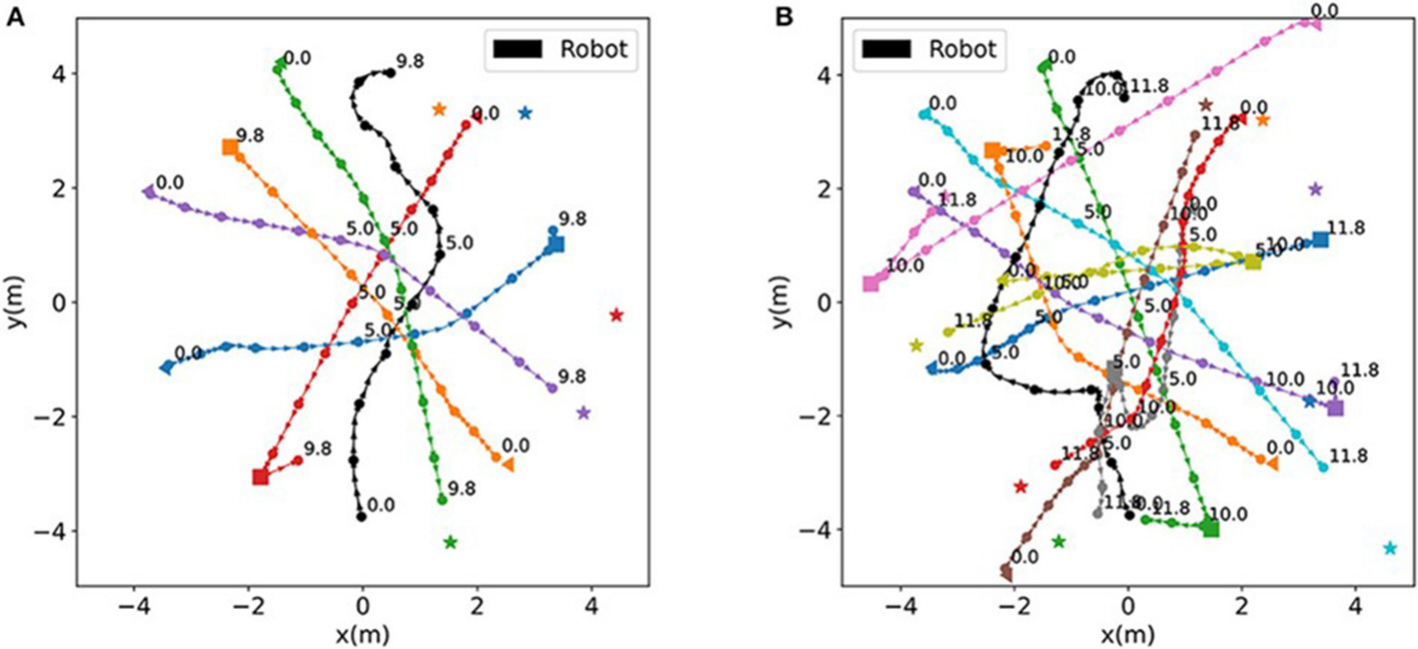
Sample trajectory maps in **(A)** a simple environment and **(B)** a complex, crowded environment.

Figure 9 shows example navigation trajectories: panel (A) illustrates a simple scenario and panel (B) illustrates a complex one. The black curve marks the robot's path (with start and end markers), while colored curves trace individual human agents. This illustrates how the robot weaves through moving obstacles.

**Figure 10:**
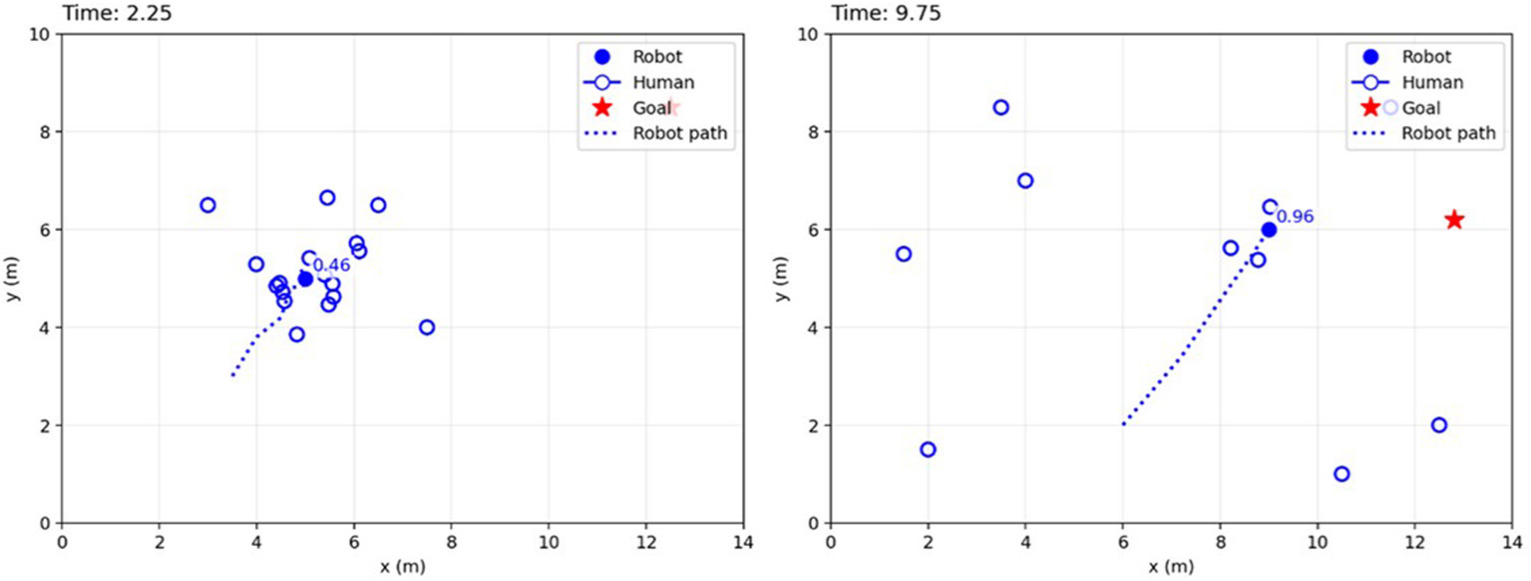
Safety evaluation visualization. **(A)** Low safety score (0.46): many humans nearby, so the robot moves slowly. **(B)** High safety score (0.96): fewer nearby humans, and the robot moves faster toward its goal.

Figure 10 depicts the real-time safety evaluation: in (A), the robot's safety score is 0.46 (many nearby pedestrians), and it moves cautiously, whereas in (B), the score is 0.96 (fewer nearby pedestrians), and the robot moves more directly. These qualitative maps emphasize that the proposed method maintains larger safety margins around the robot.

### 6.1 Overall performance

The proposed method achieves the highest success rate and the lowest constraint and proximity violations while matching or improving time-to-goal relative to baselines. Table 6 summarizes the overall aggregate metrics of the proposed approach.

**Table 6:** Aggregate overall performance metrics (mean ± SD).

**Method**	**Success rate (%)**	**Time-to-goal (s)**	**Constraint violations (per episode)**	**Proximity violations (per episode)**	**Minimum clearance (m)**	**Near-miss rate (% steps ≤ 0.3 m)**
ORCA	84.7 ± 2.1	12.10 ± 1.00	0.120 ± 0.050	0.62 ± 0.20	0.42 ± 0.09	8.4 ± 2.1
DWA	88.3 ± 1.8	11.50 ± 0.90	0.080 ± 0.030	0.48 ± 0.16	0.47 ± 0.08	6.7 ± 1.8
PPO (vanilla)	90.4 ± 1.5	10.80 ± 0.70	0.050 ± 0.020	0.31 ± 0.11	0.51 ± 0.07	4.3 ± 1.2
Proposed	98.0 ± 0.8	10.50 ± 0.60	0.020 ± 0.010	0.19 ± 0.08	0.66 ± 0.06	2.6 ± 0.9

Relative to PPO, collisions and constraint violations drop by ~60% (0.05 → 0.02/ep), the near-miss rate is approximately halved, and minimum clearance increases by ~0.15 m while maintaining competitive time-to-goal.

### 6.2 Ablation study

Table 7 isolates the contributions of forecasting, the CBF shield, and horizon and risk modeling. Removing either forecasting or shielding degrades safety and reliability; shortening the forecast horizon moderately affects performance; disabling risk awareness increases proximity incursions. All ablations keep the same transformer-based forecasting backbone; systematic variation of the predictor itself (e.g., graph-based or GAN-based models such as Trajectron++ and SocialGAN-RL) is left for future work, enabled by the modular interface between the forecaster and the Safe-RL + CBF stack.

**Table 7:** Ablation study of the proposed navigation stack (mean ± SD).

**Variant**	**Success rate (%)**	**Time-to-goal (s)**	**Constraint violations (per episode)**	**Proximity violations (per episode)**	**Minimum clearance (m)**
Full (proposed)	98.0 ± 0.8	10.50 ± 0.60	0.020 ± 0.010	0.19 ± 0.08	0.66 ± 0.06
No forecast	92.1 ± 1.2	11.20 ± 0.80	0.050 ± 0.020	0.34 ± 0.12	0.54 ± 0.07
No shield (CBF off)	90.2 ± 1.4	10.60 ± 0.70	0.110 ± 0.040	0.41 ± 0.15	0.49 ± 0.08
Short horizon (H/2)	95.8 ± 1.0	10.80 ± 0.60	0.040 ± 0.020	0.26 ± 0.10	0.60 ± 0.07
Risk-blind (no CVaR)	96.4 ± 1.0	10.60 ± 0.60	0.030 ± 0.010	0.23 ± 0.09	0.61 ± 0.07

## 7 Discussion

The results indicate that coupling transformer-based human-motion forecasting with a Safe-RL policy and a CBF shield yields meaningful safety gains without sacrificing efficiency. Compared with ORCA, DWA, and vanilla PPO, the proposed method consistently increases the success rate, increases the minimum clearance, and reduces both constraint and proximity violations (Section 6). The ablation study (Table 3) attributes most of the gains to (i) anticipatory information from forecasting—critical in doorways and cross-flows—and (ii) the run-time CBF projection, which eliminates a large fraction of residual unsafe actions while minimally perturbing the nominal policy. Robustness analyses further show stable performance under increased crowd density and injected sensor–network latency (Table 8), suggesting that uncertainty-aware forecasting and latency-compensated safety checking are complementary.

**Table 8:** Uncertainty-aware forecasting and latency-compensated safety evaluation.

**Condition**	**Method**	**Success (%)**	**Time-to-goal (s)**	**Near-miss rate (% steps ≤ 0.3 m)**	**Minimum clearance (m)**
Low density	ORCA	92.3 ± 1.2	10.8 ± 0.6	4.1 ± 1.0	0.55 ± 0.07
	DWA	94.0 ± 1.1	10.4 ± 0.5	3.6 ± 0.9	0.58 ± 0.06
	PPO	96.0 ± 0.9	10.0 ± 0.5	2.8 ± 0.8	0.61 ± 0.06
	Proposed	99.0 ± 0.5	9.8 ± 0.4	1.9 ± 0.6	0.70 ± 0.05
Medium density	ORCA	86.7 ± 1.8	12.3 ± 0.8	8.0 ± 1.9	0.47 ± 0.08
	DWA	89.5 ± 1.6	11.9 ± 0.8	6.3 ± 1.6	0.50 ± 0.07
	PPO	91.1 ± 1.4	11.2 ± 0.7	4.6 ± 1.3	0.54 ± 0.07
	Proposed	98.2 ± 0.9	10.7 ± 0.6	2.9 ± 1.0	0.66 ± 0.06
High density	ORCA	75.1 ± 2.5	13.7 ± 1.0	13.2 ± 2.8	0.38 ± 0.10
	DWA	79.8 ± 2.2	13.2 ± 0.9	10.9 ± 2.4	0.41 ± 0.09
	PPO	83.2 ± 1.9	12.6 ± 0.8	8.1 ± 2.0	0.46 ± 0.08
	Proposed	95.6 ± 1.1	11.6 ± 0.7	4.8 ± 1.6	0.60 ± 0.07
100 ms latency	ORCA	83.4 ± 2.0	12.6 ± 0.9	9.0 ± 2.1	0.45 ± 0.08
	DWA	86.2 ± 1.8	12.0 ± 0.8	7.2 ± 1.8	0.48 ± 0.08
	PPO	88.5 ± 1.6	11.3 ± 0.7	5.5 ± 1.5	0.52 ± 0.07
	Proposed	96.9 ± 0.9	10.8 ± 0.6	3.5 ± 1.1	0.63 ± 0.06
300 ms latency	ORCA	76.2 ± 2.4	13.2 ± 1.0	11.6 ± 2.5	0.41 ± 0.09
	DWA	80.0 ± 2.2	12.8 ± 0.9	9.8 ± 2.2	0.44 ± 0.09
	PPO	82.7 ± 2.0	12.1 ± 0.8	7.3 ± 1.9	0.49 ± 0.08
	Proposed	94.0 ± 1.2	11.3 ± 0.7	4.6 ± 1.4	0.58 ± 0.07

**Figure 11:**
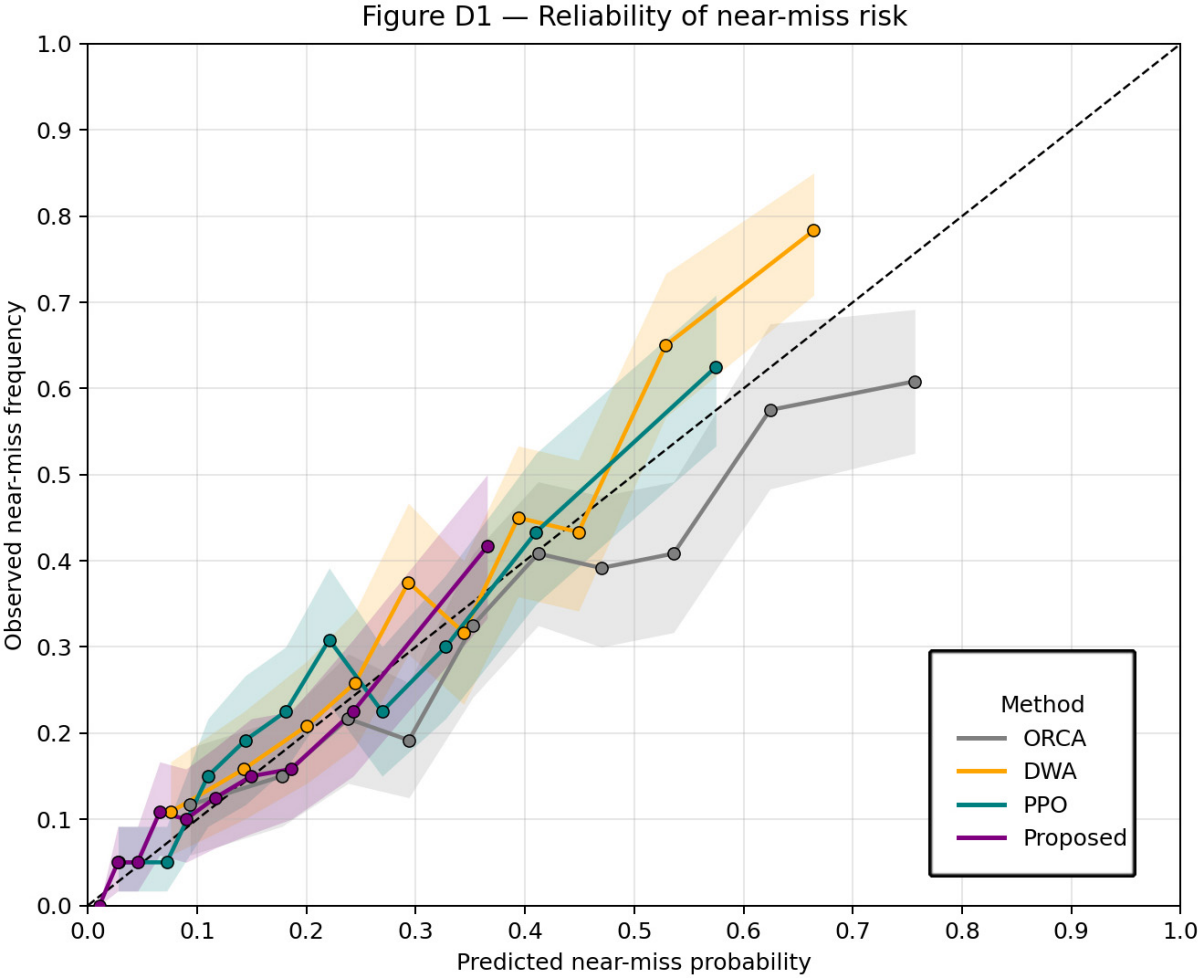
Reliability of near-miss risk.

To assess probabilistic calibration, Figure 11 plots reliability diagrams comparing predicted near-miss risk against empirical near-miss frequency across methods. Curves closer to the diagonal indicate better calibration; the proposed method tracks the diagonal more closely than PPO and DWA, explaining its stronger risk-aware decisions.

Although our calibrated Gaussian mixture forecaster used in this study does not provide the finite-sample coverage guarantees of conformal prediction, it offers continuous densities and covariances that couple naturally to the chance-constrained CBF construction and to the risk features consumed by the Safe-RL policy, all within a 20–30 ms control budget. Conformal predictors for multi-agent trajectories would typically yield set-valued forecast tubes and require an additional calibration loop, and mapping such sets into differentiable risk features and real-time CBF constraints is non-trivial in our embedded setting. For these reasons, this study adopts a lightweight parametric forecaster with explicit *post hoc* calibration. It considers conformalized trajectory prediction a promising direction for extensions targeting stronger theoretical uncertainty guarantees.

### 7.1 Mechanisms and interpretation

**Figure 12:**
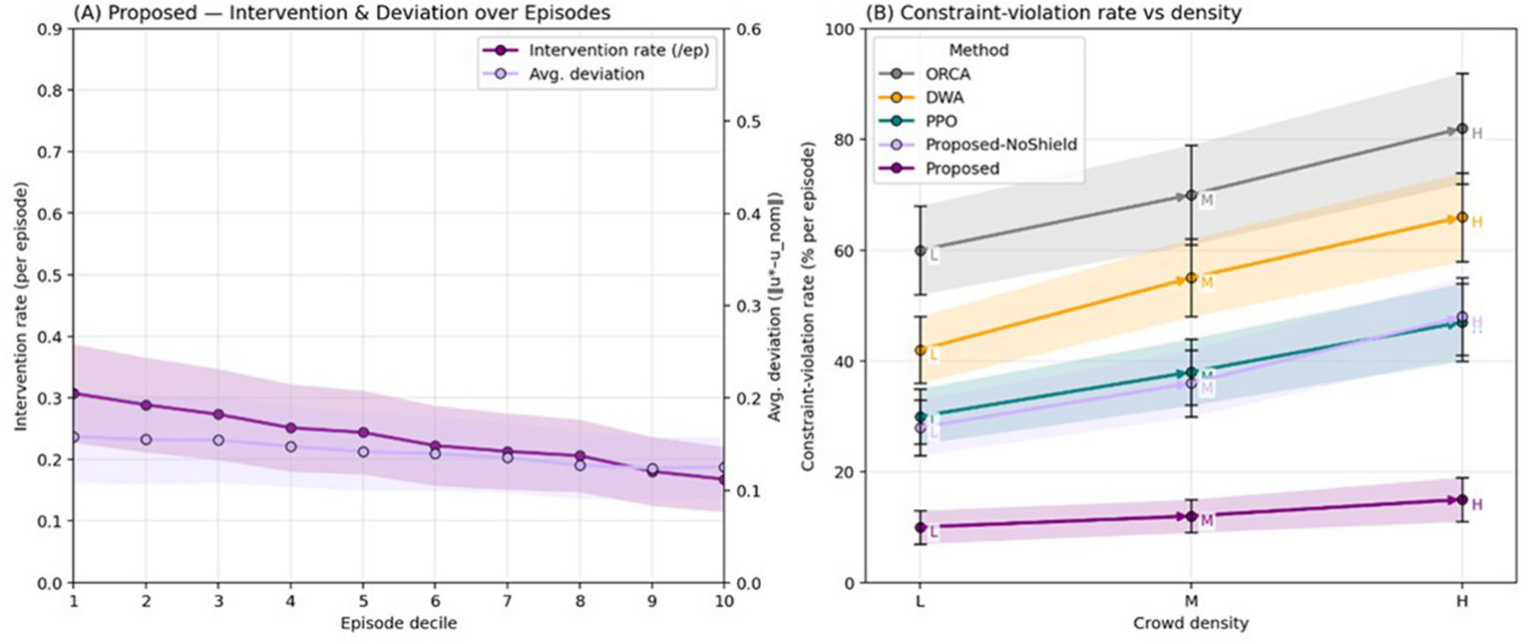
Shield interventions vs. violations (lines + shaded bands): **(A)** proposed intervention and deviation over episodes; **(B)** constraint-violation rate vs. density.

Forecasting supplies short-horizon, uncertainty-calibrated occupancy that the policy uses to pre-emptively adjust speed and path, thereby avoiding last-second evasions that often trigger near-misses in baseline methods. The CBF shield then provides formal safety at execution time; intervention logs show low activation rates but high protective value (Figure 12). Importantly, time-to-goal remains competitive because interventions are sparse and small in magnitude, so efficiency is largely governed by the learned policy rather than by conservative fail-safes.

The ablation results also clarify the impact of specific design choices. Shortening the forecast horizon or disabling calibration increases shield interventions and produces more hesitant, stop-and-go motion, while removing the CVaR head (“risk-blind”) leads to higher rates of near-miss events despite similar average success. Keeping the CBF shield modular, rather than absorbing it into the policy network, simplifies verification and debugging and preserves formal safety guarantees, at the cost of a small fraction of projected actions in dense interactions.

### 7.2 Robustness to density and latency

Performance degrades gracefully with pedestrian density and with injected latencies of 100–300 ms (Table 9). Baseline methods experience a sharp rise in near-miss events under these conditions. In contrast, the proposed method maintains lower violation rates due to forecasting-aware risk features and delay-aware CBF evaluation. Failure analysis shows that most remaining errors arise from abrupt group flow changes (i.e., forecast drift) and rare cases of CBF infeasibility near tight bottlenecks; both effects are reduced but not eliminated by the safety shield.

**Table 9:** Failure modes for each navigation stack in the crowded corridor and four-bed ward scenarios (medium pedestrian density, 100 ms latency).

**Failure mode**	**ORCA**	**DWA**	**PPO**	**Proposed**	**Proposed-NoShield**
***Scenario context: corridor** + **four-bed ward; medium pedestrian density; 100 ms latency; values reported as percentage of episodes per failure mode***.					
Collision with a moving human	6.1	4.3	2.7	1.1	3.9
Collision with a static object	1.8	1.2	0.9	0.4	1.1
Stall/timeout (no progress)	4.4	3.7	2.6	1.9	2.2
Oscillation near the doorway	3.5	2.9	1.8	0.9	1.7
Forecast drift → late avoidance	–	–	1.4	0.7	1.8
CBF infeasibility (tight bottleneck)	–	–	–	0.3	–

Values are percentages of episodes terminated due to each failure mode.

### 7.3 Practical implications and limitations

For clinical deployments, higher clearance and fewer near-misses translate to improved perceived safety and reduced staff burden. The modularity of the pipeline allows drop-in replacement of the forecaster or shield to suit hospital layouts and compute budgets. Four limitations remain: (i) sensitivity to sharp, collective flow reversals that can temporarily miscalibrate forecasts; (ii) occasional CBF infeasibility in extremely tight spaces; (iii) the evaluation fixes a single transformer-based forecasting backbone rather than benchmarking alternative predictors (e.g., Trajectron++- or SocialGAN-RL–style models), so the effect of predictor choice on closed-loop safety and efficiency is not yet quantified; and (iv) the shield induces a mild distribution shift because the environment executes $a_{t}^{sh}$ while the policy samples $a_{t}^{\pi }$. In practice, this shift is bounded by the intervention norm ∥δ*a*_*t*_∥, which remains small in the evaluated regimes, but a full theoretical treatment of this off-policy effect remains an open direction for future work. These issues may be mitigated by (a) on-device online calibration of the forecaster, (b) conservative fallback braking with verified invariance, (c) operator-intent overlays that enable quick authority handover in edge cases, and (d) future experiments that integrate multiple forecasting backbones into the same Safe-RL + CBF framework.

Although the simulations instantiate hospital-like corridors and four-bed wards, these layouts and traffic patterns closely resemble those found in many LTC homes, where telepresence robots are increasingly explored to support remote visitation and care-partner engagement. We, therefore, expect the anticipatory behaviors learned here to transfer to LTC settings, while acknowledging that dedicated real-world LTC evaluations remain an important next step. To mitigate “shield myopia,” the critic is conditioned on the augmented input (*s*_*t*_, δ*a*_*t*_, λ_*t*_), so that value estimates depend on how often and how strongly the shield intervenes, while a regularizer on ∥δ*a*_*t*_∥ encourages the actor to move toward low-intervention regimes and internalize safety within the policy.

### 7.4 Future directions

Promising extensions include world-model–based MPC for longer-horizon planning under partial observability, multi-agent coordination informed by staff wayfinding policies, and prospective trials with real hospital traffic to validate social-comfort outcomes. Another important direction is the systematic comparison of different human-motion prediction backbones (e.g., Trajectron++-like, SocialGAN-RL, and diffusion-based crowd forecasters) within the same Safe-RL + CBF architecture, to characterize how forecasting model choice trades off safety margins, efficiency, and computational load in clinical layouts. Integration with shared-autonomy overlays for operator intent may further reduce rare stalls without compromising safety.

## 8 Conclusion

This study presented an integrated forecast → policy → safety-filter pipeline in which transformer-based human-motion forecasting augments a risk-aware Safe-RL policy, while a discrete-time CBF shield enforces run-time safety for telepresence co-navigation in hospital wards. The formulation explicitly addresses partial observability, dynamic human flows, and sensing/network delays, operationalizing anticipatory perception through short-horizon, uncertainty-calibrated occupancy features and latency-compensated safety projection—precisely the gap identified in prior work, where perception, learning, and formal safety have rarely been fused for TPRs in clinical environments. Quantitatively, the approach outperformed three strong baselines (ORCA, DWA, and PPO). Relative to PPO, constraint violations fell by 60.0% (0.05 → 0.02 per episode), proximity violations by 38.7% (0.31 → 0.19), and near-miss rate by 39.5% (4.3% → 2.6% of steps ≤ 0.3 m), while time-to-goal improved by 2.8% (10.8 → 10.5 s). Success rate increased by 7.6 percentage points (90.4% → 98.0%), and minimum clearance increased by 0.15 m (0.51 → 0.66 m). Against ORCA, improvements were larger (e.g., 83.3% fewer constraint violations, 69.0% lower near-miss rate, and 13.2% faster time-to-goal). These results confirm that anticipatory forecasting, coupled with a certified safety layer, materially enhances safety without sacrificing efficiency—addressing the core clinical requirement for reliable, human-compatible telepresence mobility. Ablation evidence clarifies the mechanism of benefit. Removing forecasting reduced success by 5.9 percentage points and raised constraint violations by 150% and proximity violations by 78.9%; turning off the CBF shield reduced success by 7.8 percentage points and increased constraint violations by 450% and proximity violations by 115.8%. Shortening the forecast horizon doubled the number of constraint violations and increased proximity incursions by 36.8%, underscoring the value of short-horizon anticipation. Sensitivity analyses showed graceful degradation under crowding and latency: at 300 ms injected delay, the method still achieved 94.0% success vs. 82.7% (PPO) and 80.0% (DWA), with consistently larger clearances. Compared to existing studies focused on either reactive social navigation, learning-only policies, or control-only certificates, the proposed integration demonstrably closes the identified research gap by jointly leveraging (i) transformer forecasting for anticipation, (ii) Safe-RL for adaptability, and (iii) a CBF shield for formal run-time guarantees in people-dense clinical layouts. In practice, these gains translate into fewer near-misses, larger comfort distances, and maintained throughput—benefits that can reduce staff burden and increase acceptability in wards. Future work can extend this foundation by adapting on-device forecasters to mitigate rare forecast drift during abrupt flow reversals, implementing conservative, verified fallbacks for tight bottlenecks, and providing operator-intent overlays for rapid authority handover. Longer-horizon world-model–MPC and prospective studies in live hospital workflows are natural next steps to convert the demonstrated ~39–83% safety reductions (depending on baseline and metric) and ~3–13% efficiency gains into sustained clinical impact. Taken together, these findings suggest that transformer-based forecasting, Safe-RL, and CBF shields form a viable architectural template for anticipatory, safety-constrained navigation in people-dense clinical layouts. Although the results are obtained in simulation benchmarks, they surface design principles—short-horizon forecasting, explicit uncertainty handling, and modular safety layers—that can guide future deployments of telepresence systems in hospital and LTC wards. More broadly, this study illustrates how forecast-aware shared autonomy can help bridge the gap between high-performance learning-based navigation and the stringent safety and acceptability requirements of healthcare environments.

## Data Availability

The datasets presented in this study can be found in online repositories. The names of the repository/repositories and accession number(s) can be found below: https://doi.org/10.5281/zenodo.17034737.
